# Delayed diagnosis as a barrier to Zero Leprosy: Lived experiences from Pakistan

**DOI:** 10.1371/journal.pgph.0007184

**Published:** 2026-08-27

**Authors:** Anil Fastenau, Rhea Brüggemann, Abdul Salam, Charlotte Nehring, Maresa Neuerer, Hamideh Ebrahimi, Alexandra Asboeck, Matthew Willis, Sophie C. W. Unterkircher, Fabian Schlumberger, Heleen Neeltje Willemijn Duighuisen

**Affiliations:** 1 Marie Adelaide Leprosy Center, Karachi, Pakistan; 2 Department of Global Health, Institute of Public Health and Nursing Research, University of Bremen, Bremen, Germany; 3 German Leprosy and Tuberculosis Relief Association (DAHW), Wuerzburg, Germany; 4 Heidelberg Institute of Global Health, University of Heidelberg, Heidelberg, Germany; 5 Faculty of Medicine, Institute for Occupational, Social, and Environmental Medicine, Friedrich Alexander University Erlangen-Nuremberg, Erlangen, Germany; 6 Lahore School of Nursing, University of Lahore, Lahore, Pakistan; 7 RG Implementation Research (AG Fusco), Bernard Nocht Institute for Tropical Medicine, Hamburg, Germany; 8 Rawalpindi Leprosy Hospital, Rawalpindi, Pakistan; 9 Department of Public Health, Institute of Tropical Medicine, Antwerp, Belgium; 10 University of Antwerp, Antwerp, Belgium; 11 Care and Public Health Institute (CAPHRI), Maastricht University, Maastricht, The Netherlands; SUPA71 Co., Ltd, THAILAND

## Abstract

Despite progress in leprosy control, delayed diagnosis remains a barrier to achieving Zero Leprosy, particularly in low-endemic settings. Diagnostic delay not only sustains hidden transmission but also leads to preventable disability and lifelong consequences for affected individuals. Pakistan, although classified as low endemic, continues to report a high proportion of new leprosy cases presenting with grade 2 disability (G2D), indicating delays in case detection. This study explored the reasons for delayed leprosy diagnosis in Pakistan and participant-suggested strategies to reduce delay, from the perspective of persons presenting with visible disability. A qualitative study was conducted using semi-structured interviews with persons affected by leprosy who presented with G2D at diagnosis. Participants were recruited through leprosy service providers in Pakistan. Interviews explored care-seeking pathways, experiences with the health system, stigma, and perceived barriers to timely diagnosis. Audio-recorded interviews were transcribed and analysed using thematic analysis, with attention to the interpreter-mediated nature of some participant accounts. Participants described prolonged diagnostic pathways marked by repeated healthcare visits, misdiagnosis, inappropriate treatment, and referrals between multiple providers before receiving a leprosy diagnosis. Limited awareness of leprosy among the public and frontline healthcare workers was a contributor to delay. Stigma emerged as both a cause and a consequence of delayed diagnosis, influencing disclosure, care-seeking behaviour, and social interactions. Structural barriers, fragmented service provision, financial constraints, and the absence of a national leprosy control programme, further compounded delays, resulting in diagnosis only after the onset of visible deformities. Diagnostic delay in Pakistan is driven by intersecting individual, social, and health system factors and remains an obstacle to preventing disability and interrupting transmission. From a Zero Leprosy perspective, reducing diagnostic delay is a clinical, programmatic and ethical imperativ. Strengthening awareness, improving health worker training, decentralising diagnostic services, and integrating person-centred psychosocial support can advance early detection and disability prevention.

## Introduction

Leprosy, a neglected tropical disease caused by *Mycobacterium leprae*, remains a leading cause of preventable disability worldwide, particularly in low-resource settings [1]. Despite substantial progress in treatment and control, leprosy continues to pose a global public health challenge, with nearly 200,000 new cases reported worldwide each year [2]. Although multidrug therapy (MDT) has markedly reduced prevalence, delayed diagnosis remains a key barrier to leprosy elimination [3,4]. Diagnostic delay not only prolongs transmission but also increases the risk of irreversible nerve damage and visible deformities, clinically categorized as grade 2 disability (G2D) under WHO guidelines [5]. Therefore, active case finding and timely diagnosis remain central priorities for advancing leprosy control and elimination [4].

In line with the global ambition of Zero Leprosy, which seeks to achieve zero transmission, zero disability, and zero discrimination, timely diagnosis and prevention of disability have become central priorities [2]. In low-endemic settings, diagnostic delay represents a major obstacle to this goal, as it sustains hidden transmission and leads to preventable, lifelong disability.

Pakistan, while classified as a low-endemic country since reaching elimination targets in 1996, still reports over 250 new cases annually [6]. In leprosy control, elimination as a public health problem has historically been defined by WHO as a registered prevalence of less than 1 case per 10,000 population, a threshold Pakistan reached in 1996. Alarmingly, a disproportionately high proportion of these cases present with G2D at the time of diagnosis, reflecting a concerning trend of delayed case detection [7]. In Karachi alone, 38% of newly diagnosed cases in 2022 were classified with G2D [8]. Prior quantitative and mixed-methods investigations have identified a median delay of two years between symptom onset and diagnosis, with even longer delays observed in males, older individuals, and those with multibacillary (MB) disease [8,9].

Qualitative research in Pakistan has further contextualized these delays, revealing complex structural and sociocultural drivers [10]. These include limited public awareness and persistent stigma, insufficient clinical training among general healthcare providers [10], and an absence of a national leprosy control program, with service provision largely dependent on two non-governmental organizations (NGOs) [6,7].

Persons affected by leprosy, particularly those presenting with visible deformities, frequently report experiences of neglect, misdiagnosis, poverty-related access barriers, and social exclusion, all of which exacerbate delays in seeking and receiving appropriate care [5,11].

While expert perspectives and health system analyses have shed light on structural and provider-level challenges [10], there remains a significant knowledge gap regarding the lived experiences of persons affected by visible deformities due to leprosy. Understanding their narratives is essential to inform person-centered strategies that can reduce diagnostic delays, enhance early detection, and improve disability prevention [11]. This study aims to address this gap by exploring both the reasons for delayed leprosy diagnosis in Pakistan and participant-suggested strategies to reduce diagnostic delay, from the perspective of individuals presenting with G2D, using a qualitative approach.

## Methodology

### Ethics statement

This study was conducted in full accordance with the principles of the Declaration of Helsinki, as adopted by the World Medical Association, which provides ethical guidance for medical research involving human participants. All procedures involving human subjects were designed to respect the rights, safety, dignity, and well-being of participants. The study was approved by the Ethical Review Committee of the Marie Adelaide Leprosy Centre (MAC/ERC/2024/03) in May 2024. Participants were provided with an information sheet translated into Urdu outlining the purpose of the study. Written and verbally informed consent was obtained from all participants prior to the interviews. Participants indicated their consent by either signing their name or providing a thumbprint. Interviews were conducted confidentially, and identifying details were anonymized. All data is pseudonymized and stored on a secure, password protected device and will be deleted after five years.

### Study design

This study employed a qualitative approach to explore the lived experiences of persons affected by leprosy who presented with visible deformities (G2D) at diagnosis. The study aimed to capture in-depth insights into personal, social, and health system-related factors contributing to diagnostic delays. The study was conducted by the Marie Adelaide Leprosy Centre (MALC) and the German Leprosy and Tuberculosis Relief Association (GLRA) in February 2025.

### Study setting and study population

The study was conducted in Karachi, Pakistan, the country’s most populous city and a recognized hotspot for leprosy detection [8]. Participants were purposively selected among individuals registered at the MALC, a national NGO and referral center for leprosy care. MALC is one of the main non-governmental providers of leprosy diagnosis, treatment, disability care, rehabilitation, and follow-up services in Pakistan, and functions as a major referral centre in Karachi and surrounding areas. Persons with suspected or confirmed leprosy may reach MALC directly, through referral from public or private healthcare providers, or through MALC subcentres and outreach networks. However, not all persons affected by leprosy in Pakistan are necessarily managed by MALC, and the study population therefore reflects individuals connected to MALC services rather than all persons affected by leprosy nationally. Most participants were associated with the MALC headquarters, while others were contacted through MALC subcentres in peripheral areas to include perspectives from both urban and rural settings. Participants were identified through MALC facility records and through staff at MALC headquarters and selected subcentres who were familiar with persons affected by leprosy receiving or having previously received care. Potential participants were screened against the eligibility criteria and approached by MALC staff either during routine contact with services or by telephone where contact details were available. Older cases were identified through existing MALC records and follow-up networks, as many persons affected by leprosy remain known to MALC through disability care, rehabilitation, ulcer care, or social support services even after completion of MDT. Interested individuals were provided with study information and invited to participate voluntarily. Eligibility criteria included: confirmed diagnosis of leprosy, documented or clinically recognized G2D, age 18 years or older, ability and willingness to provide informed consent, and willingness to discuss their diagnostic pathway in an in-depth interview. Individuals were excluded if they declined participation, were unable to complete the interview, or if the interview was terminated prematurely. Due to the relatively low number of G2D cases within the study setting, it was decided to not set any restrictions on the year of diagnosis. Participants received detailed information and signed informed consent forms. This study included five female and 14 male participants between 18 and 72 years with different educational backgrounds. Seven participants were currently employed, four mentioned that they had worked before, one was a student (Table 1). Sampling was purposive and aimed to capture variation in gender, age, place of residence, educational background, employment status, and time since diagnosis, while focusing specifically on persons with G2D. Recruitment continued until no new major codes, categories, or themes emerged during the analysis.

**Table 1:** Participants’ sociodemographic characteristics.

Participant	Age	Place of residence	Gender	Employment	Educational Attainment Level
P1	35	urban, Karachi	m	yes	MBA degree
P2	49	urban, Karachi	m	yes	10th grade
P3	65	urban, Karachi	m	no	no
P4	50	homeless, Karachi	m	no	no
P5	18	urban, Karachi	f	no	no
P6	70	rural, Badin (Sindh)	m	no	no
P7	72	urban, Karachi	m	yes	2 years
P8	54	Karachi, Lyari town	m	yes	middle school (6/7th grade)
P9	50	urban, Karachi	m	no	6 months
P11	60	Karachi, Surjani town	m	yes	10 years
P12	27	rural, Summar Kandani Malir	f	no	5th grade, primary school
P13	21	rural, Summar Kandani Malir	m	no	bachelor’s degree
P14	64	rural, Summar Kandani Malir	m	no	2 years
P15	45	rural	f	no	no
P16	69	urban, Landhi number 3, Karachi town	m	no	8 years
P17	54	Malir	f	yes	intermediate (1st year of college)
P18	28	urban, Orangi number 1	m	no	5th grade, primary school
P20	45	urban, Karachi, Orangi town	f	no	2 years
P21	48	urban, Karachi, Orangi town	m	yes	intermediate (1st year of college)

*P10 and P19 were excluded due to premature termination of the interview

### Data collection and analysis

Twenty-one in-depth, semi-structured interviews were conducted with individuals affected by G2D. However, two interviews (P10 and P19) were terminated prematurely, thus only 19 interviews were analyzed. An interview guide was developed drawing on prior studies on diagnostic delay and adapted for cultural relevance (S1 Text). Open-ended questions explored participants’ routes to diagnosis including symptom recognition, health-seeking behavior, barriers to accessing care, and experiences with healthcare providers. These standardized questions were reviewed and revised beforehand. Interviews were conducted in a private setting and lasted between 40 and 100 minutes, with an average duration of 75 minutes. During the interviews, either a MALC or GLRA staff member familiar with the topic and the cultural and linguistic context translated between English and Urdu. Translation was non-simultaneous; thus, some participants’ statements were summarized rather than reproduced literally. Prior to the onset of the interview, participants answered a sociodemographic questionnaire including place of residence, age, gender, education and employment. All interviews were audio recorded with an external device while the researchers took notes to capture non-verbal cues.

Audio recordings of the English interview dialogue and the interpreter-mediated Urdu responses were transcribed verbatim where direct English speech was available. Because translation during interviews was non-simultaneous, some Urdu responses were rendered into English as summarized translations rather than literal word-for-word translations. We therefore treated quoted material as translated participant accounts rather than verbatim Urdu quotations. To enhance accuracy, interviews were conducted with MALC/GLRA staff familiar with the linguistic, cultural, and clinical context, field notes were taken during interviews to capture non-verbal cues and contextual meaning, and transcripts were checked against recordings and notes during analysis. Formal back-translation and participant member-checking were not conducted, which is acknowledged as a limitation. Transcripts were analyzed using thematic content analysis supported by ATLAS.ti 24 software [12]. Expanding on previously developed deductive codes, an inductive coding strategy was applied to identify recurring themes and subthemes. Data collection and analysis proceeded iteratively, allowing the research team to assess the depth and recurrence of emerging themes. By the nineteenth completed interview, later interviews largely reinforced previously identified themes, with no new major codes, categories, or themes emerging. Coding and interpretation were reviewed within the research team, and differences were discussed until agreement was reached. Analytical notes and successive versions of the coding framework were maintained to support transparency and consistency. Quotes illustrating results were lightly edited for readability by removing filler words, repetitions, and stutters, without altering meaning. Where interpreter-rendered statements were expressed indirectly, they were converted into first-person form only when this improved readability and when the intended meaning was clear from the recording and field notes. Some participants shifted between first-person accounts and general statements about “people” or “they,” particularly when discussing stigma, health-seeking behaviour, or community awareness. Such statements were interpreted as perceived norms or collective observations unless they were explicitly linked to the participant’s own experience. The main themes that were found included 1) characteristics of the disease, 2) personal barriers, 3) social barriers, and 4) health system-related barriers. As a fifth theme 5) recommendations on reducing diagnostic delays have been gathered from the participants’ perspective. After thematic analysis was completed, the various barriers for delay were distinguished between individual-level, community-level, and health system-level barriers. For this study, diagnostic delay was defined as the period between the participant’s first recalled sign or symptom compatible with leprosy and the point at which a leprosy diagnosis was received from a healthcare provider. Delay duration was estimated from participant self-report and, where available, checked against clinical or registration records. Because symptom onset and early consultations were often difficult to recall precisely, delay estimates were treated as approximate and used primarily to contextualize participants’ narratives rather than as systematically measured epidemiological outcomes. Throughout the manuscript, “diagnostic pathway” refers to the sequence of symptom recognition, care-seeking, consultations, referrals, and eventual diagnosis, while “barriers” refer to factors that contributed to prolonging this pathway. Below, the results are presented accordingly.

For clarity, the final thematic framework is summarized in Table 2. The theme “characteristics of the disease” was treated as an individual-level barrier in the Results because participants described how painless, slowly progressive, and initially ambiguous symptoms influenced their recognition of illness and timing of care-seeking.

**Table 2:** Thematic framework of barriers and participant-suggested strategies.

Analytical level	Main theme	Subthemes
Individual level	Characteristics of the disease	Painless symptoms; slow progression; delayed recognition; symptoms noticed by others
Individual level	Personal barriers	Limited knowledge; low perceived severity; self-medication; fear; financial and opportunity costs
Community level	Social barriers	Stigma; label-based stigma; non-disclosure; gender norms
Health system level	Health system-related barriers	Limited clinical expertise; misdiagnosis; weak referral pathways; poor communication; affordability and accessibility
Cross-cutting	Participant-suggested strategies	Awareness campaigns; involvement of persons affected by leprosy; health worker training; referral guidance; outreach and active case finding

### Reflexivity

The research team comprised researchers and practitioners with backgrounds in leprosy care, global health, qualitative research, and public health, and interviews were conducted with the support of MALC/GLRA staff familiar with the clinical, cultural, and linguistic context. The researchers had no prior personal relationship with the participants. Their contextual knowledge facilitated trust, interpretation of local terminology, and understanding of care pathways. However, the team recognized that its professional and institutional affiliations with leprosy services, particularly MALC/GLRA, could have influenced how participants described their experiences of services, referrals, and healthcare providers. To minimize this risk, participants were informed that participation was voluntary, their responses would remain confidential, and their decision to participate would not affect their access to care or support. During analysis, the team reflected on how its professional roles and prior knowledge could shape interpretation and critically discussed emerging themes to distinguish participants’ accounts from researcher assumptions.

## Results

Based on participant self-report and, where available, clinical or registration information, all participants described a delay between first recalled symptoms and diagnosis, ranging approximately from six months to 30 years. Some individuals first noticed symptoms during childhood, leaving their parents responsible for managing their healthcare. Because participants were diagnosed across different time periods, their narratives should be understood as cross-generational accounts rather than as descriptions of a single, uniform health system context. Where relevant, we therefore interpreted barriers as either persistent across time, such as limited leprosy awareness and stigma, or as potentially shaped by the historical context of diagnosis, such as availability of services, referral networks, and clinical training. Moreover, generational differences emerged due to variation in the timing of diagnosis: while some participants had been diagnosed many years earlier, others had received their diagnosis more recently. Based on the interviews, reasons for diagnostic delay occurred on the individual-, community-, and health system-level, which are often intertwined.

### Individual-level barriers

Various challenges to early diagnosis were encountered on the individual level ranging from low health seeking behavior due to a lack of knowledge about leprosy or financial constraints to difficulties in recognizing symptoms because of the ambiguous presentation of the disease Reflecting on broader community norms and barriers to early care-seeking, one participant summarized several individual-level factors that emerged across the interviews:

*“Most of the people they don’t know about signs and symptoms. If they know about everything, they’ll definitely go to show or seek treatment as soon as possible. And the people also have some financial issues. The health-seeking behavior is also not good. I mean, they are careless, you can say, about their health, most of the people.”* (P17, estimated delay of 3 years)

### Characteristics of the disease

Leprosy typically progresses slowly, often beginning with invisible non-specific symptoms - such as inconspicuous skin patches located on the back - without pain or functional impairment [13]. Several participants reported becoming aware about symptoms only after someone else noticed, or incidentally during consultations for other health concerns. Many participants did not seek medical attention as long as the disease did not interfere with their daily activities, work, nor cause discomfort.

*“It’s not a barrier in any work, or any task to perform, so they don’t want to go to doctor. (...) The people always seek help when they feel, or they face, any problem in their daily routine, or daily task, or they feel some pain, so then they seek for help. “* (P16, estimated delay of 4 years)

Participants shared different stories in which they eventually recognized their symptoms, particularly the loss of sensation. Some recounted discovering burns on their hands while cooking or injuries on their feet after stepping on sharp objects without realizing *(“… I was not feeling pain, but I could see. This I remember.” (P5, estimated delay of 5 years*). Overall, those findings demonstrate overlooked symptoms due to the absence of pain delaying the moment when illness becomes actionable, shifting health-seeking to later complications.

### Limited knowledge and awareness

The most frequently mentioned individual-level factor was limited knowledge about, on the one hand, the existence of leprosy and, on the other hand, its signs, symptoms and progression. Aligning with general limited health literacy, many participants had never heard of leprosy prior to their diagnosis. Even among those who knew about the disease, *“there was no awareness about it”* (P8, estimated delay of 3 years). Participants commonly lacked knowledge of signs and symptoms and therefore could not recognize symptoms or attribute those to leprosy.

*“People don’t have the information and education and awareness about the disease. So, people should know. Because if people don’t know they don’t know what to look for.”* (P2, estimated delay of 2.5 years)

Participants often misattributed symptoms to other conditions, such as diabetes or allergies, reflecting widespread misinformation about the disease’s etiology. Some participants believed their symptoms would heal by themselves, revealing limited awareness regarding the progression and potential consequences of the disease. As a result, most participants did not grasp the seriousness of the disease.

In addition to widespread limited knowledge about the disease and signs and symptoms, participants also lacked understanding of what actions to take once symptoms appeared.

*“There is no clear pathway also for people who have leprosy. I’ll give an example. If that person had leprosy, he doesn’t know where to go. Often, again, he or she doesn’t know what it is. And even if they know their symptoms, where to go? And then they keep spreading it further. Not only for themselves, but there is also ongoing transmission.”* (P2, estimated delay of 2.5 years)

Recalled feelings of confusion and disbelief, being unsure of what was happening to them or how to respond, distinguishes the results into knowledge of the disease, as well as knowledge of what to do.

### Limited healthcare (seeking) behavior

Many participants highlighted a generally low level of healthcare-seeking behavior within the Pakistani society.

*“It is a general attitude. Even today, people avoid, avoid to go to the doctor until that disease turns into a severe condition.”* (P8, estimated delay of 3 years)

Many admitted personal responsibility for delayed care, blaming themselves rather than criticizing the healthcare system. Some attributed disease complications to their own “laziness” or “carelessness”, emphasizing the responsibility of persons affected by leprosy for taking care of one’s health and seeking medical attention when needed. Some participants reported ignoring or neglecting symptoms as long as they did not cause discomfort or interfere with daily activities, assuming that the symptoms would not worsen over time. For example:

“*But the reason I did not go to any doctor, because it never bothered me, and there was not a problem in my work or health, so I just saw that.”* (P2, estimated delay of 2.5 years)

Consequently, healthcare was often sought only once symptoms became severe or affected work, mobility, or daily functioning. In the absence of pain, early signs were commonly underestimated, managed through self-medication, or acted upon only after another person noticed symptoms and encouraged care-seeking. Additionally, diverse life circumstances, such as homelessness or drug addiction, further delayed participants healthcare-seeking behavior.

*“I can’t say that I was careless, or my priority was not my health, it was just earning money. I was homeless. “* (P4, estimated delay of 30 years)

*“I was just taking that heroin and drugs so the main reason (…) because I was always in ‘Jannat’, I was always in this high state. So that’s why I didn’t care.”* (P9, estimated delay of 10 years)

Fear of amputation emerged as an additional barrier to timely healthcare-seeking behavior (“… they will cut your leg because your leg is too bad.” (P4, estimated delay of 30 years). Other participants were afraid isolation after being admitted to the hospital, describing it as similar to imprisonment.

Others recounted previous negative experiences within healthcare facilities or the fear of costs, wishing to avoid unnecessary expenditures. Additionally, few mentioned irregular or interrupted treatment. One participant noted that seeing all other persons affected by leprosy at the hospital having visible deformities led him to believe that treatment would be ineffective:

*I also had some misunderstanding, I, at the time, I was young, I thought these people were also at that hospital, but still they have disability, so if I go there, it won’t help, and then maybe I will get disability, because everyone at the hospital had a disability.”* (P3, estimated delay of 10-15 years)

Costs were elaborated on with regards to prioritizing expenses for food and shelter, or, with multiple ill members within the family, other diseases were perceived as more severe. Moreover, opportunity costs emerged when needing to take a day off to go to a health facility.

*“One thing was that I didn’t know it’s leprosy but it’s also the matter of resources. So, for me, I had a job and if you have to go out for a doctor, you have to take a day off and it will not be paid. So, I did not have these resources and also financial. So, they are not only in terms of money but time because you can’t just take a leave and say I am sick. So, you have to quit a day which is not paid and also you need money. So, that’s why I said oh it’s not serious. So, I will only go with something very serious.”* (P2, estimated delay of 2.5 years)

Overall, healthcare-seeking behavior was limited through an interplay of avoidance, merely partial symptom disclosure, reliance on self-medication, or needed external prompting by others. It is based on different underlying categories of fear and financial factors.

### Community-level barriers

In addition to lack of knowledge and awareness among individuals, there exists a broader deficiency in general health literacy within the Pakistani society, representing one of the most significant barriers to early diagnosis. Public information and education about leprosy - its existence, manifestations and progression – remain limited, resulting in stigmatization of persons affected by leprosy accompanied by gender-specific challenges [11].

### Stigmatization

Among the 19 participants, seven mentioned experiences of stigma or expressed the fear of stigmatization and social exclusion. They described apprehension about receiving a leprosy diagnosis, anticipating that it might lead to a change in people’s attitudes towards them. In most cases, the leprosy-related stigma was rooted in misinformation, rumors or ancient legends. Stigma appeared to be particularly present in certain communities, often resulting in non-disclosure of the condition and delayed healthcare-seeking. One participant recounted that her mother feared her husband would abandon the family if he discovered her daughter’s leprosy diagnosis. Another participant explained the following:

*“I was worried about what will happen to me because at that time the fear was very much, the stigma was very much in our country against this disease. At home the family members, mothers, brothers, they have no change of attitude. But the relatives, when they came to know that I have leprosy, then their attitude has changed towards me.”* (P8, estimated delay of 3 years)

Notably, the stigma is frequently rather associated with the name of the disease than with its actual symptoms. All participants in the interviews referred to leprosy using different local terms, most commonly *jezam* and *kor*. While *jezam* corresponds to the literal Urdu translation of leprosy, *kor* carries a highly negative connotation and is strongly associated with stigma. It is widely perceived as referring to a “very bad” disease, which led many participants to reject this label for their own condition, despite having little or no knowledge of leprosy itself. Consequently, this label-based stigma leads to or reinforces actions towards non-disclosure and delayed care.

### Gender-specific barriers

One female participant highlighted a general lack of attention to women’s problems, diseases or health concerns. She explained that women are typically not permitted to visit a doctor by themselves, they need to be accompanied by a male relative such as father, brother or husband, who must be available or able to take leave from work. It demonstrates, women sometimes must wait until a male family member finds time to accompany them, leading to delays in seeking care. This barrier, however, may be less pronounced among elder women. Cultural norms further restrict women’s ability to receive adequate medical examinations, as it is considered inappropriate for women to undress in front of male healthcare workers. Given the shortage of female healthcare staff, many female persons affected by leprosy are therefore not fully examined and remain undiagnosed. Nonetheless, perceptions of gender-specific barriers varied among female participants. While some women emphasized these challenges, others reported that they did not experience additional barriers to accessing healthcare due to their gender.

*“From woman perspective there’s also accessibility issue because as a woman I cannot come alone even if I would have money, I have to wait the brother (...) for any woman the brother or father (…) they have to take a leave from work, they have to take her. (…) I have to wait whenever my brother has time, money for the traveling and also time because he has to not go to job.”* (P5, estimated delay of 5 years)

### Health system-level barriers

Nearly all participants reported having several consultations with different kinds of healthcare providers before leprosy was considered, a clinical diagnosis was made, or they were referred to an appropriate healthcare facility. Many described moving in and out of various healthcare facilities over several years, enduring a long diagnostic pathway that consumed significant time, financial resources, and trust in the healthcare system.

### Limited leprosy expertise among healthcare workers

A reason for the multitude of consults that some participants had to endure is found in the unfamiliarity with leprosy among doctors and healthcare workers.

*“But I remember, and that makes me sad, that actually, I was always going to doctor, but nobody was able to diagnose me.”* (P3, estimated delay of 10-15 years)

Most of the participants reported that several of the doctors they consulted neither knew nor thought of leprosy as a possible diagnosis. Some were unaware of the disease’s existence; others did not recognize characteristic signs and symptoms. According to the participants, most doctors lacked the necessary expertise in diagnosis, particularly clinical diagnosis, even among specialists. However, they pointed out that doctors ought to be responsible for a correct diagnosis.

Due to limited leprosy expertise, most doctors only treated the visible symptoms presented by persons affected by leprosy, such as dressing ulcers or prescribing medication, without investigating the underlying cause, performing full-body clinical examinations, or requesting laboratory confirmation.

*“The second reason is that the majority at that time, now its maybe changed, the majority of the doctors and dermatologists, they don’t have any training in leprosy. They don’t know. So, they are not able to clinically or generally, they are not able to diagnose. So, the lack of expertise of leprosy or the knowledge of leprosy is lacking among the healthcare staff and the doctors and dermatologists.”* (P1, estimated delay of 2-3 years)

Participants commonly described either receiving no diagnosis or being misdiagnosed with conditions such as diabetes, cancer, allergic reactions, or typhoid fever. Leprosy was often considered only after severe signs, such as clawed hands, had developed, while earlier symptoms were overlooked. As a result, many participants received diagnosis and treatment only after irreversible complications had already occurred.

### Limited quality of leprosy care

About one third of the participants raised concerns about the quality and qualifications of healthcare providers. They criticized the prevalence of unqualified or unprofessional practitioners, particularly in low-income communities, whose legitimacy was often uncertain, similar to faith healers or the practice of traditional medicine or black magic. In some cases, participants suspected that certain doctors prioritized financial gain over patient care and deliberately avoided referring persons affected by leprosy to a specialized leprosy facility.

*“They didn’t know, they didn’t think of leprosy or even they don’t know about leprosy. I don’t think that a doctor won’t know about leprosy, but they didn’t think of leprosy. If he knew that I have leprosy, but he will not refer me because he is getting money from me. So, he will not refer me to the doctor.”* (P14, estimated delay of 5 years)

Even when a leprosy diagnosis was eventually made, participants described poor communication from healthcare providers. They reported being unaware of what was happening, what procedures would be performed, or even the exact results of their diagnostic tests.

“*I felt that they did not explain me, or I did not understand so clearly that really if I would not take the treatment, I can get to disability. I did not understand.”* (P3, estimated delay of 10-15 years)

Furthermore, participants noted limited availability of treatment, as persons affected by leprosy could not be treated in all facilities, but only in specialized hospitals.

### Lack of a clear referral system

Half of the participants expressed limited knowledge regarding specialized leprosy facilities among both healthcare providers and persons affected by leprosy. Many participants had never heard of MALC before. Consequently, this lack of awareness contributes to inadequate referral practices, reflecting a weak referral system in Pakistan, particularly Karachi. According to our participants, numerous doctors and healthcare workers failed to refer persons affected by leprosy to specialized facilities once they were unable to diagnose properly. Many did not know where to direct persons affected by leprosy, thus participants had to visit multiple healthcare facilities only to get referred again before being send to the appropriate center.

“*(…) everyone they do (…) my family and me did the same, but we went to the nearest one. And when they told us to go to Jinnah hospital, so we went there. Then Jinnah told us to go to Skin Hospital, we went there. And when the Skin Hospital told us to come to MALC, we went there.”* (P16, estimated delay of 4 years)

The experienced stepwise referrals without clear guidance demonstrate how the navigation burden is placed on persons affected by leprosy.

### Limited trust in the healthcare system

Participants repeatedly expressed limited trust in the healthcare system, including treatment, medication and vaccination. This distrust extended throughout the community or even society. To mitigate uncertainties, individuals preferred being treated by someone familiar, who was easily approachable. Nevertheless, participants generally were rather hesitant or reluctant to engage with healthcare services, as illustrated by the following:

“*Every time the doctors or the babas or hakeems they will say ‘trust us, take this, you will be fine’, I will do the same, I will not get fine and again it starts again. So, my family and I we all were tired and then there was just lack of trust because it was not helping, there was no improvement and, in this time, things were getting worse and worse*” (P5, estimated delay of 5 years)

### Affordability and accessibility

Despite the availability of free leprosy treatment, all previous consultations prior to reaching a specialized leprosy facility where they received an accurate diagnosis each associated with costs. Moreover, nearly half of the participants reported they were unaware that free treatment options existed. Expenditures further increased once persons affected by leprosy paid for ineffective treatment or unnecessary medication. Consequently, affordability emerged as a recurring barrier to healthcare including timely diagnosis, making it a “*luxury to go to a skin doctor”* (P2, estimated delay of 2.5 years). Financial constraints not only decided about the frequency of seeking medical care but also determined the quality of healthcare providers they could afford. Participants perceived that considerably competent doctors, particularly those in larger hospitals, charged higher fees while affordable ones lacked adequate qualifications.

In addition to affordability, accessibility emerged as a closely related challenge. Many participants needed to travel great distances to reach healthcare facilities, particularly hospitals or specialists, as such services were either not available everywhere or expensive. In many low-income communities, affordable high-quality treatment options were limited or entirely absent, while hospitals were located far away. Most individuals therefore required transport to cover those distances imposing an additional financial burden.

“*We need to use two buses. It was far from our residence. And it was very difficult to pay for this two. And we have to spend a lot on these two buses.*” (P17, estimated delay of 3 years)

“*That the main hospitals, even you don’t have to pay there, but the accessibility is an issue. Because when I went there, at that time, I spent more than 300 rupees. And it’s a big amount at that time.”* (P11, estimated delay of 6 months)

Further accessibility barriers arose from overcrowded healthcare facilities, especially those offering free treatment options, resulting in long waiting times. Participants recounted spending hours at facilities only to remain unattended or to be referred elsewhere.

To summarize, participants expressed dissatisfaction with what they perceived as a weak healthcare system, characterized by the absence of leprosy expertise among healthcare workers and a clear and efficient pathway towards diagnosis and treatment.

### Participant-suggested strategies to reduce diagnostic delay

After exploring the participants’ perspectives on reasons contributing to the diagnostic delay, they were asked for their suggestions to reduce the time between the onset of symptoms and a clinical diagnosis.

### Awareness campaigns

It was highlighted that participants would have appreciated it if they already possessed knowledge about leprosy.

*“I wish that I knew before that about this disease and the signs and symptoms, so I could seek help earlier.”* (P20, estimated delay of 5 years)

In accordance with Participant 20’s statement, most participants emphasized the urgent need to raise awareness about leprosy, so that the general public becomes informed not only about the disease’s existence but also about its signs, symptoms and available healthcare services. The following participant elaborated on this:

*“This is a disease, like, in beginning, the patient or the person does not feel anything, so he does not go to the doctor, so if with the awareness, we can tell the people that okay, if you have this type of symptoms and you don’t treat it, so maybe you will face problems or you will get deformed, so this is his responsibility, but then if he notices something, he will definitely, and he should go to a doctor, and then the doctor or the health facility, it is their responsibility that they should be trained, they should know about everything, and then they should be able to diagnose such a disease.”* (P16, estimated delay of 6 months)

Participants suggested using social media, television, billboards, schools, and community gatherings to raise awareness of early signs, such as skin patches and loss of sensation, and to demonstrate simple self-examination techniques. They emphasized that campaigns should be visual, engaging, and practical, clearly communicating that leprosy is curable, treatment is free, and care is available at specific facilities.

They suggested showing how the unproblematic could become problematic would encourage individuals to seek care, fostering a sense of personal responsibility for health once they realize the disease’s seriousness. Participants further recommended teaching leprosy in schools or at community gatherings, delivered by trained professionals or community leaders, MALC team members, or persons affected by leprosy who have completed their treatment successfully.

### Involvement of persons affected by leprosy

More than half of the participants underscored their own responsibility in tackling leprosy in Pakistan.

*“There should be a responsibility of those who are diagnosed with this, and they should tell the other people that okay this is disease and how you should.”* (P9, estimated delay of 10 years)

They suggested the active involvement of persons affected by leprosy who had completed treatment as “agents of change” that could share their experiences, raise awareness, motivate and encourage others to seek diagnosis and treatment. As individuals affected by leprosy themselves, they are uniquely positioned to spread accurate information within communities about signs, symptoms and facilities while simultaneously contributing to the reduction of stigma and fear. To take on these roles effectively, participants emphasized the need for training in communication skills and leprosy-specific knowledge. In addition, 13 of 19 participants expressed willingness to identify symptoms, doing a first “examination” and refer potential cases. Further, their visibility would reinforce the message that leprosy is curable and that those affected are just “normal people”.

### Strengthening healthcare system

While most participants focused on individual responsibility, the involvement of persons affected by leprosy, and strategies to spread information, some also highlighted the need for improvements within the healthcare system. As one participant noted: “*Every doctor should be responsible.”* (P11, estimated delay of 6 months). Another one suggested that doctors and healthcare workers need to be educated not only about leprosy but also trained in symptom recognition as well as conducting proper clinical examinations and diagnoses.

Additionally, participants emphasized the importance of clear communication between doctors and persons affected by leprosy regarding diagnosis and treatment, as well as improved awareness about the referral system. They underlined that healthcare facilities need to be aware of MALC and its services:

“*You can tell all the people (…) about this hospital. So, the people can come and seek treatment. And also tell the other hospitals, where you have relations to, tell them that this type of patient can be sent here”* (P20, estimated delay of 5 years)

Other participants suggested, based on their lived experiences, establishing a dedicated hotline or guidance number for healthcare workers who were uncertain about diagnosis or referral pathways, as well as an information desk within hospitals to guide persons with suspected leprosy regarding procedures and subsequent steps. Some participants further proposed scaling up outreach programs, skin camps and active case finding initiatives, particularly in hotspot areas through the healthcare sector. Further, they mentioned the need for more qualified healthcare centers within villages to ensure accessible care nearby. One female participant specifically emphasized the importance of increasing the number of female healthcare workers to overcome gender specific barriers to leprosy diagnosis. Finally, one participant called for greater government oversight in verifying the qualifications of doctors and clinics, ensuring that persons affected by leprosy receive safe and competent medical care.

## Discussion

This qualitative study provides in-depth insight into the multifaceted reasons for delayed leprosy diagnosis in Pakistan from the perspectives of persons affected by grade 2 disability (G2D). Our findings demonstrate that diagnostic delay is not a single-point failure but rather the cumulative outcome of intersecting individual-, community-, and health system-level barriers. Importantly, these delays have implications that extend far beyond individual morbidity: they directly undermine efforts to interrupt *Mycobacterium leprae* transmission and therefore constitute a critical obstacle to achieving Zero Leprosy in Pakistan [4,7].

### Diagnostic delay as a driver of ongoing transmission

Although this qualitative study did not measure transmission directly, participants’ accounts of prolonged undiagnosed periods, delayed referral, and lack of awareness can be interpreted in light of existing evidence on leprosy transmission and disability as indicators of missed opportunities for early detection and prevention.

A central implication of delayed diagnosis, often underemphasized in programmatic discourse, is its role in sustaining leprosy transmission within communities. Individuals with undiagnosed leprosy may remain infectious for years [1], particularly in contexts where household crowding, poverty, and prolonged close contact are common. Participants’ narratives revealed delays ranging from months to decades, during which time they continued normal social and occupational activities without awareness of their condition. These prolonged undiagnosed periods may create repeated opportunities for transmission to household members, neighbors, and coworkers, potentially contributing to the reservoir of undetected disease. This finding is highly relevant in Pakistan, a country officially categorized as low-endemic but characterized by persistent detection of new cases with high disability proportions [8,9]. The consistently high proportion of G2D among new diagnoses is not merely a clinical indicator of late presentation; it is a sentinel marker of systematic failure to interrupt transmission chains early. As long as diagnostic delay remains unaddressed, Zero Leprosy targets, particularly zero transmission and zero child leprosy, are unlikely to be achieved [14].

### Delay, disability, and the paradox of “low endemic” settings

Our findings reinforce the paradox observed in many low-endemic countries: while overall case numbers decline, diagnostic delay increases, resulting in higher disability rates at diagnosis [15]. Reduced disease incidence leads to diminishing public awareness, declining clinical suspicion among healthcare workers, and erosion of leprosy expertise within the general health system [15,16]. Participants repeatedly described prolonged diagnostic journeys marked by misdiagnosis, symptomatic treatment without etiological investigation, and failure to conduct basic sensory examinations.

This phenomenon creates a self-reinforcing cycle. As leprosy becomes perceived as a “rare disease,” it is deprioritized in medical education and routine clinical practice [16,17]. Consequently, early signs, particularly painless skin patches and sensory loss, go unrecognized until irreversible nerve damage occurs. From a Zero Leprosy perspective, this represents a critical misalignment: elimination strategies increasingly depend on earlier detection, not reduced vigilance. For health systems in low-endemic countries, these findings point to several concrete programmatic priorities: maintaining leprosy competence within general health services, embedding simple sensory testing and skin examination into frontline and dermatology care, establishing clear referral pathways to specialized centres, and using G2D at diagnosis as a routine indicator of diagnostic timeliness. Low case numbers should therefore not lead to reduced investment; rather, they require targeted systems that preserve clinical suspicion and ensure rapid referral when suggestive symptoms appear.

### Stigma, non-disclosure, and hidden transmission

Stigma emerged as both a cause and a consequence of delayed diagnosis, consistent with findings from other countries [18–21]. Participants described fear-driven non-disclosure, avoidance of healthcare facilities, and concealment of symptoms, often driven more by the stigmatized label of leprosy than by physical manifestations. This concealment is particularly consequential for transmission dynamics, as it delays not only diagnosis but also contact tracing and post-exposure prophylaxis.

In the context of Pakistan, where leprosy services are largely NGO-driven and not fully integrated into national primary healthcare, stigma interacts with structural invisibility [11]. Individuals may live for years with undiagnosed leprosy while remaining embedded in social networks that facilitate transmission. Thus, stigma functions not only as a psychosocial burden but as a biological risk amplifier, prolonging infectiousness and undermining prevention efforts.

Interpreted through a stigma framework, these findings show how leprosy-related stigma operates not only at the interpersonal level, through fear of rejection and altered family or community relationships, but also at the anticipatory and internalized levels, where fear of being labelled discourages disclosure and delays care-seeking. The local terminology used for leprosy, particularly highly stigmatized labels such as *kor*, illustrates how language itself can become a mechanism of exclusion. In this sense, stigma does not merely follow diagnosis; it actively shapes the pathway to diagnosis by making symptoms socially risky to disclose.

### Missed opportunities for prevention and post-exposure prophylaxis

Delayed diagnosis also represents missed opportunities to deploy proven preventive interventions, particularly contact tracing and single-dose rifampicin post-exposure prophylaxis (SDR-PEP) [22]. Early case detection is the entry point for preventive strategies; without it, household and social contacts remain unidentified and unprotected [4]. Several participants explicitly noted that they were unaware of where to seek care or were referred only after advanced disability had developed by which point further transmission may already have occurred.

In Zero Leprosy roadmaps, early detection is not an end in itself but the gateway to prevention [4]. Reducing diagnostic delay, therefore, is not only about preventing disability but about strategically interrupting transmission before it propagates to the next generation of cases [14].

### Gender, marginalization, and delayed interruption of transmission

Gender-specific barriers identified in this study further complicate transmission dynamics. Women’s restricted mobility, dependence on male relatives for healthcare access, and limited availability of female healthcare providers can delay diagnosis in a population already underrepresented in case detection data [21]. Such delays may result in prolonged exposure of household contacts, reinforcing intergenerational transmission.

Similarly, participants experiencing homelessness, substance use, or extreme poverty described diagnostic delays spanning decades. These structurally marginalized populations often fall outside routine health surveillance, creating “blind spots” in elimination efforts. From a Zero Leprosy lens, these groups are not peripheral but central: failing to reach them sustains low-level endemicity despite overall progress.

Our sample size does not allow conclusions about whether diagnostic delays were systematically longer among women than men. In this study, some male participants also reported long delays, particularly where symptoms were painless, livelihood pressures were high, or trust in healthcare was low. However, female participants described additional gendered mechanisms that may shape delay, including restrictions on independent mobility, dependence on male relatives for transport or permission, and discomfort with examination by male healthcare workers. These gender norms may also encourage concealment of symptoms, especially where a diagnosis could affect marriage, family relationships, or social standing. Future studies with larger female samples should examine whether these mechanisms translate into measurable gender differences in delay duration, disability at diagnosis, and access to referral services.

These findings can also be understood through the lens of structural violence, as delayed diagnosis was not only the result of individual choices but of constrained options shaped by poverty, gender norms, unstable housing, dependence on daily wages, and uneven access to competent healthcare. Participants’ narratives show that “delay” often reflects a lack of realistic alternatives: seeking care may require transport costs, unpaid time away from work, accompaniment by male relatives, or repeated navigation of poorly connected services. Framing diagnostic delay in this way shifts responsibility away from individuals alone and highlights the social and institutional conditions that make late diagnosis more likely.

### Implications for zero leprosy in Pakistan

The health system barriers identified in this study operate at several levels. At the primary care level, early symptoms were often missed or treated symptomatically without sensory testing or suspicion of leprosy. At the specialist level, participants reported that even dermatology or hospital services sometimes failed to recognize leprosy until advanced disability had developed. Operationally, weak referral communication, poor counselling of persons affected by leprosy, and limited awareness of specialized services delayed movement through the care pathway. Structurally, the absence of a fully integrated national leprosy programme and reliance on NGO-led services limited the routine visibility of leprosy within primary healthcare and surveillance systems. Distinguishing these levels is important because each requires different interventions: clinical training, referral tools, navigation of persons affected by leprosy, and stronger government stewardship.

Taken together, our findings position diagnostic delay as a critical and potentially modifiable barrier to achieving Zero Leprosy in Pakistan [4,6,7]. Addressing delay of leprosy diagnosis requires coordinated action across awareness generation, health system strengthening, and service decentralization [17,23]. The findings can be directly mapped onto the three pillars of the Global Leprosy Strategy and Zero Leprosy agenda: zero transmission, zero disability, and zero discrimination. Delayed diagnosis undermines zero transmission by allowing undiagnosed individuals to remain in close contact with households and communities before treatment begins. It undermines zero disability because missed early symptoms and repeated misdiagnosis allow preventable nerve damage to progress to visible deformity. Finally, it undermines zero discrimination because stigma, especially label-based stigma around terms such as *kor* and *jezam*, discourages disclosure, delays care-seeking, and reinforces social exclusion. Addressing diagnostic delay therefore requires more than improved clinical detection; it requires integrated action across prevention, disability care, and stigma reduction.

Person-centered strategies, including the meaningful involvement of persons affected by leprosy as community educators and navigators, appear particularly promising [24,25]. Participants themselves articulated a clear understanding that early recognition and timely referral could have altered their disease trajectories. Empowering these voices aligns with rights-based approaches and enhances the credibility of elimination messages within communities [25,26]. The NGO-led structure of leprosy services in Pakistan has been essential for maintaining specialized expertise, treatment access, and continuity of care in a low-endemic context. However, reliance on NGOs alone also creates vulnerabilities for Zero Leprosy efforts, particularly if leprosy expertise remains concentrated in specialized centres rather than embedded within the national primary healthcare system. Achieving Zero Leprosy by 2030 will require stronger government stewardship, including formal integration of leprosy into primary healthcare, routine training of frontline health workers, standardized referral pathways, inclusion of leprosy indicators in national surveillance systems, and sustained financing for contact tracing, disability prevention, rehabilitation, and stigma reduction. NGOs such as MALC can continue to provide technical leadership and specialist support, but government ownership is necessary to ensure nationwide coverage, accountability, and long-term sustainability.

### Strengths of lived-experience perspectives

A key strength of this study lies in its exclusive focus on persons affected by leprosy with visible deformities, whose experiences illuminate the end-stage consequences of systemic delay. While prior research has examined diagnostic delays from provider or programmatic perspectives, these narratives underscore the human cost of late detection and reveal critical gaps that quantitative surveillance alone cannot capture. Importantly, they also demonstrate that individuals affected by leprosy possess valuable insights into feasible, culturally acceptable solutions.

### Limitations

The study’s qualitative design enabled an in-depth analysis of the perceptions and experiences of persons affected by leprosy, but several limitations should be acknowledged. First, the study presents the perspectives of persons affected by leprosy only. While this provides a valuable complement to previous regional research that explored expert perspectives on diagnostic delay [10], it does not include the views of healthcare workers, family members, caregivers, or programme managers. In addition, because the study focused specifically on persons who had already developed G2D, the findings are likely to overrepresent more severe and prolonged diagnostic pathways. Individuals diagnosed earlier, before visible disability developed, may have experienced different barriers, shorter care-seeking pathways, or more effective referral processes. The focus on G2D is therefore valuable for understanding the consequences of delayed diagnosis, but it may bias the findings toward more extreme delay narratives and limits comparison with persons diagnosed at earlier disease stages. Accordingly, the findings may not be fully transferable to persons diagnosed before developing disability, individuals without disability, or those who have not accessed specialized leprosy services.

Second, the study relied on retrospective accounts. Some participants found it difficult to recall the precise timing of symptom onset, first consultations, referrals, and diagnosis, particularly when diagnostic pathways extended over many years or decades. Reported delay durations should therefore be interpreted as approximate and contextual rather than as exact epidemiological measures. In addition, delay estimates may have been influenced by memory reconstruction, whereby participants interpreted earlier symptoms, healthcare encounters, or turning points through the lens of their later diagnosis and disability. The inclusion of participants diagnosed across different periods also means that some narratives reflect health system contexts that may differ from the current situation in Pakistan. Changes over time in leprosy awareness, service availability, NGO outreach, referral networks, and clinical training may have influenced the relevance of some reported experiences. However, the recurrence of similar barriers among participants diagnosed more recently suggests that limited awareness, weak referral pathways, stigma, and insufficient leprosy expertise remain persistent challenges.

Third, although non-simultaneous interpretation during interviews enabled a better flow of conversation, it may have increased the risk of translation errors and reduced the linguistic richness of the data. Idiomatic expressions, emotional emphasis, and culturally specific phrasing may not have been fully captured in the English rendering. This is particularly relevant in qualitative research, where the exact wording of participants’ narratives can carry important analytical meaning. Although the involvement of experienced staff and the use of field notes helped preserve contextual meaning, the absence of formal back-translation or member-checking limits our ability to fully verify the accuracy and nuance of translated accounts.

Fourth, the sample was gender-skewed, with only five female participants compared with fourteen male participants. Although several important gender-specific barriers emerged, including women’s dependence on male relatives for travel and limited access to female healthcare workers, the small number of female participants restricts the depth and transferability of these findings. Future studies should purposively recruit larger numbers of women affected by leprosy, including women with disability, to better understand the intersection of gender, mobility restrictions, stigma, and delayed diagnosis in Pakistan.

Lastly, children were not included in the study population, although diagnostic delay among children is particularly important for disability prevention and for identifying recent transmission. Future research should therefore include children and adolescents affected by leprosy, as well as caregivers, to better understand paediatric diagnostic pathways and missed opportunities for early detection.

## Recommendations

Based on the lived experiences of persons affected by leprosy with visible deformities, this study identifies several priority actions that correspond directly to the main themes of the analysis. Limited symptom recognition and uncertainty about where to seek care support the need for public awareness and self-examination messages. Repeated misdiagnosis and missed sensory testing point to the need for stronger clinical capacity within general and dermatology services. Weak referral experiences and limited knowledge of MALC services support clearer referral pathways and navigation of persons affected by leprosy. Stigma, label-based fear, and non-disclosure justify the meaningful involvement of persons affected by leprosy in awareness and stigma-reduction activities. Finally, gendered mobility restrictions, poverty, homelessness, and substance use highlight the need for inclusive and gender-sensitive service delivery. Addressing these interconnected barriers requires coordinated interventions across community, health system, and policy levels [27–29].

### Public awareness and symptom recognition

Targeted awareness initiatives should be expanded, particularly in identified hotspot areas [30], to improve recognition of early leprosy signs such as hypopigmented skin patches and loss of sensation. Campaigns should emphasize that leprosy is curable, treatment is free of charge, and early diagnosis prevents disability and transmission. Information should be disseminated through culturally appropriate channels, including community gatherings, schools, mass media, and digital platforms, with clear guidance on where and how to seek care.

### Actively involve persons affected by leprosy

The meaningful involvement of persons affected by leprosy should be institutionalized within awareness, case-finding, and stigma-reduction activities [31]. As trusted members of their communities, individuals who have completed treatment are uniquely positioned to promote early healthcare seeking, counter misinformation, and reduce fear associated with the disease. Structured training and support should be provided to enable their safe and effective engagement as peer educators, community advocates, and navigators within the health system [32].

### Strengthen clinical capacity within the general health system

Given the declining exposure to leprosy in low-endemic settings, sustained investment in clinical capacity building is essential [15,27]. Leprosy-specific training should be integrated into curricula for physicians, nurses, and allied health workers, with a focus on clinical diagnosis, sensory testing, and early referral. Particular emphasis should be placed on training frontline providers and dermatology services, where persons affected by leprosy often first present.

### Improve referral pathways

Clear, well-communicated referral pathways between primary healthcare facilities and specialized leprosy centres are urgently needed. All healthcare providers should be informed about existing referral centres and diagnostic services. In addition, referral pathways should explicitly include access to mental health services in order to address the substantial psychosocial burden, stigma, and mental health needs associated with leprosy [33].

### Enhance gender-sensitive and inclusive service delivery

Strategies to reduce diagnostic delay must explicitly address gender-specific and structural barriers. Increasing the availability of trained female healthcare workers, particularly in primary care and outreach settings, may improve access for women. Targeted approaches are also required to reach marginalized groups, including individuals experiencing homelessness, substance use, or extreme poverty, who are often excluded from routine health services and surveillance. Future research and programmatic monitoring should specifically include women affected by leprosy, as gendered barriers may remain hidden when women are underrepresented in both surveillance data and qualitative research.

### Link early diagnosis to prevention and transmission interruption

Early case detection should be systematically linked to contact tracing and preventive interventions, including single-dose rifampicin post-exposure prophylaxis. Reducing diagnostic delay is a prerequisite for the effective implementation of preventive strategies and for interrupting transmission chains. Monitoring trends in grade 2 disability among newly diagnosed cases should remain a key programmatic indicator of diagnostic timeliness and health system performance.

Future quantitative and mixed-methods studies should build on these qualitative findings by measuring the relative contribution of identified delay factors, including symptom recognition, first provider contact, referral failures, gender-related access barriers, stigma, and costs. Prospective studies could link diagnostic delay intervals with G2D, household contact screening outcomes, SDR-PEP eligibility, and child case detection to better assess how delayed diagnosis affects transmission interruption. Such evidence would help prioritize interventions and define measurable indicators for Zero Leprosy implementation in low-endemic settings.

Future research should specifically include children and adolescents affected by leprosy, as child leprosy is a key indicator of recent transmission and an important marker of ongoing gaps in early detection. Understanding diagnostic pathways among children, including the role of parents, schools, primary healthcare providers, and community awareness, is essential for interrupting transmission and preventing lifelong disability from an early age.

## Conclusion

This qualitative study highlights that delayed diagnosis of leprosy in Pakistan is driven by a complex and interrelated set of individual-, community-, and health system-level barriers, as experienced by persons affected by visible deformities. Diagnostic delay is not only a determinant of irreversible disability and psychosocial harm, but also a critical factor sustaining ongoing transmission in a country officially classified as low-endemic. Our findings underscore that high proportions of grade 2 disability at diagnosis represent a sentinel indicator of missed opportunities for early detection, prevention, and transmission interruption.

Addressing diagnostic delay requires a coordinated, multi-level response that combines improved public awareness and strengthened clinical capacity within the general health system. Meaningful involvement of persons affected by leprosy emerges as a particularly powerful strategy, with the potential to enhance early symptom recognition, reduce stigma, and strengthen community trust in elimination efforts. From a Zero Leprosy perspective, reducing diagnostic delay is not merely a clinical imperative but a programmatic and ethical necessity. Without sustained investment in early case detection and person-centred approaches, leprosy will continue to circulate silently despite low national case numbers, undermining progress towards interruption of transmission by 2030. Zero leprosy in Pakistan is within reach; however, eliminating leprosy in low-endemic settings ultimately demands sustained political will to translate evidence into action [34]. Future implementation research should translate these qualitative insights into measurable indicators of diagnostic delay, referral performance, contact tracing coverage, and prevention outcomes.

## Supporting information

S1 Text Interview Guide. (DOCX)

S2 Text Sociodemographic Questionnaire and Interview Feedback. (DOCX)

## Abbreviations

G2D: Grade 2 disability
GLRA: German Leprosy and Tuberculosis Relief Association
MALC: Marie Adelaide Leprosy Centre
MB: Multibacillary
MDT: Multi-drug therapy
NGO: Non-governmental organization
SDR-PEP: Single-dose rifampicin post-exposure prophylaxis
WHO: World Health Organization
