# Supplementary material for: Delayed diagnosis as a barrier to Zero Leprosy: Lived experiences from Pakistan
Source: PLOS Glob Public Health. 2026 Aug 27;6(8):e0007184. doi: 10.1371/journal.pgph.0007184 (PMC13521354; doi:10.1371/journal.pgph.0007184)
Supplement: Supplementary file 1 [file pgph.0007184.s005.pdf]

Response to the Editor and Reviewers

Manuscript ID: PGPH-D-26-00426

Dear Editor and Reviewers,

We sincerely thank the Editor and all reviewers for their careful reassessment of our manuscript and for the constructive and encouraging feedback. We have addressed all remaining editorial and methodological points. The original comments are reproduced in full below, followed by our point-by-point responses. All changes are shown in the revised manuscript with tracked changes and incorporated into the clean manuscript.

Editorial comment: Please revise Table 1 as follows:
Heading of Table 1: Should start with capital letters; for example: Participant, Age, Place of residence, Gender, Employment, Educational. For the education should be revised to "Educational Attainment Level"

Response: Thank you for identifying this formatting issue. We revised all column headings in Table 1 so that they begin with capital letters and changed the final heading from "Education" to "Educational Attainment Level."

Reviewer #1: All comments have been addressed

Response: We sincerely thank Reviewer #1 for confirming that all previous comments have been adequately addressed and that the manuscript meets the journal's publication criteria.

Reviewer #2: This manuscript constitutes a valuable contribution to the literature on leprosy, delayed diagnosis, and strategies aimed at achieving the Zero Leprosy goal in low-endemicity settings. The study demonstrates adequate methodological rigor, high programmatic relevance, and considerable potential to inform public policies and strengthen leprosy surveillance, care, and control efforts.
The limitations inherent to the study are appropriately acknowledged and transparently discussed by the authors, and they do not undermine the robustness of the findings or the validity of the conclusions.
One of the manuscript's major strengths lies in giving voice to people affected by leprosy and reconstructing their diagnostic trajectories from the perspective of lived experience. Although previous studies have explored factors associated with delayed diagnosis from the viewpoint of specialists and healthcare professionals, this work provides important additional evidence by focusing specifically on individuals who developed visible disabilities as a consequence of delayed diagnosis.
The study is particularly relevant because it demonstrates that diagnostic delay should not be viewed solely as a clinical problem, but rather as the result of a complex interplay of individual, social, and structural factors, including low risk perception, limited awareness of the signs and symptoms of the disease, social stigma, gender inequalities, structural weaknesses within the healthcare system, insufficient professional training, and shortcomings in referral and counter-referral systems. This perspective broadens the understanding of the phenomenon, highlights its multifactorial nature, and enhances the practical value of the findings for the design, implementation, and improvement of public policies aimed at leprosy control and elimination.

Response: We are very grateful for this thoughtful and encouraging assessment. We particularly appreciate the reviewer's recognition of the value of centring the lived experiences of persons affected

by leprosy and of framing diagnostic delay as the result of interacting individual, social, and structural factors. No additional changes were requested.

Reviewer #3:

Comment 1: Resubmission – Round 2
Manuscript ID: PGPH-D-26-00426
Title: Diagnostic Delay in Leprosy in Pakistan: Lived Experiences of Persons with Grade 2 Disability
Article Type: Qualitative Research

The authors have responded thoroughly to the major concerns raised during the previous review round. The revised manuscript is substantially improved, particularly in the areas of theoretical framing, interpretation of findings, limitations, and policy relevance. The manuscript now presents a coherent and compelling account of how diagnostic delay contributes not only to disability but also to ongoing transmission, thereby impeding progress toward Zero Leprosy goals. The study offers valuable insights from persons affected by leprosy and makes a meaningful contribution to global public health and NTD literature.

Most remaining issues are editorial and relate to strengthening methodological transparency and ensuring consistency in interpretation.

Major Strengths of the Revision
1. Improved Theoretical and Analytical Depth
The revised discussion now goes beyond descriptive reporting and incorporates concepts such as:
• Stigma as a multi-level phenomenon
• Structural violence
• Health system failures at multiple levels
• Transmission dynamics in low-endemic settings
These additions substantially strengthen the manuscript's contribution to global health scholarship. The discussion of stigma operating at interpersonal, anticipatory, and internalized levels is particularly effective.

2. Stronger Linkage to Zero Leprosy Framework
The manuscript now clearly positions diagnostic delay as a barrier to:
• Zero transmission
• Zero disability
• Zero discrimination
This framing enhances the policy relevance of the findings and aligns well with current global leprosy strategies.

3. Improved Limitations Section
The revised limitations section appropriately acknowledges:
• Recall bias
• Memory reconstruction
• Translation challenges
• Gender imbalance
• Focus on G2D participants only

This significantly improves the manuscript's credibility and transparency.

4. Better Alignment Between Findings and Recommendations
The recommendations are now explicitly linked to themes emerging from participant narratives, which addresses a key concern from the previous review.

Response: We sincerely thank the reviewer for this detailed and encouraging reassessment and for recognizing the substantial improvements in the manuscript’s theoretical framing, interpretation, limitations, and policy relevance.

Comment 2: 1. Reflexivity and Researcher Positionality

Although methodological reporting has improved, the manuscript still lacks sufficient discussion of researcher reflexivity.
The authors should briefly address:
• Interviewer backgrounds
• Relationship between researchers and participants
• Potential influence of professional affiliations with leprosy services
• Steps taken to minimize interpretive bias
This is particularly important in qualitative research involving stigmatized conditions.
Suggested action: Add a short paragraph within the Methods section addressing reflexivity.

Response: We agree that explicit reporting of researcher positionality is important in qualitative research, particularly when research concerns a stigmatized condition and researchers are affiliated with leprosy services. We therefore added a dedicated reflexivity subsection.

Comment 3: 2. Trustworthiness of the Analysis

The manuscript would benefit from more explicit reporting of qualitative rigor procedures.
Please clarify:
• Whether thematic saturation was assessed and how it was determined.
• Whether coding was performed independently by more than one researcher.
• How coding disagreements were resolved.
• Whether any form of peer debriefing or audit trail was maintained.
These additions would strengthen methodological transparency.

Response: Thank you for this important suggestion. We expanded the Data Collection and Analysis subsection to explain how data collection and analysis proceeded iteratively, how recurrence of themes was assessed, how coding and interpretation were reviewed within the research team, how differences were resolved, and how analytical documentation was maintained. In Methods, subsection “Data Collection and Analysis,” page 5: we clarified that by the nineteenth completed interview, later interviews largely reinforced previously identified themes and no new major codes, categories, or themes emerged. We also stated that coding and interpretation were reviewed within the research team, differences were discussed until agreement was reached, and analytical notes and successive versions of the coding framework were maintained to support transparency and consistency.

Comment 4: 3. Sampling and Transferability

The manuscript acknowledges that all participants had grade 2 disability, which is appropriate. However, a brief statement should be added to emphasize that the findings may not fully reflect the

experiences of:
• Individuals diagnosed earlier
• Persons without disability
• Individuals who have never reached specialized services
Although partly discussed in limitations, a clearer statement regarding transferability would be beneficial.

Response: We agree and have strengthened the limitations section to make the boundaries of transferability explicit.

Comment 5: 4. Language and Terminology

A few instances still use provider-centered terminology, such as:
"patients"
Consider consistently using:
"persons affected by leprosy"
throughout the manuscript to align with contemporary person-centered language in NTD research.

Response: We agree and reviewed the manuscript throughout for person-centred language. Provider-centred terminology was replaced with “persons affected by leprosy,” “participants,” or other context-appropriate wording in the authors’ narrative. The term “patient” was retained only where it appears within direct participant quotations or published reference titles, which were not altered.

Comment 6: Minor Editorial Comment: Discussion

The discussion is now comprehensive; however, a small amount of condensation could improve readability. Some sections repeat similar arguments regarding transmission and delayed diagnosis.

Response: Thank you for this helpful observation. We reviewed the Discussion and made minor edits to reduce repetition, particularly in the section linking diagnostic delay with transmission, disability, stigma, and the Zero Leprosy targets. The overall structure and interpretation were retained.

Comment 7: Minor Editorial Comment: Recommendations

The recommendations section is strong, but the proposed hotline for healthcare workers and information desk concepts could be briefly identified as participant-generated suggestions rather than evidence-based interventions.

Response: We agree and revised the relevant Results text to make clear that these ideas emerged from participants’ lived experiences and should not be interpreted as established evidence-based interventions. In Results, subsection “Strengthening healthcare system,” the text now states that participants suggested, based on their lived experiences, establishing a dedicated hotline or guidance number for healthcare workers and an information desk within hospitals.

Comment 8: Data Availability Statement

The revised data availability statement is thoughtful and appropriately balances transparency with confidentiality concerns. No further revisions required.

Response: We thank the reviewer for this assessment. No further changes were made to the Data Availability Statement.

Reviewer #4

I have carefully reviewed the revised version and the authors' responses to the reviewers' comments. The authors have adequately addressed the concerns raised in the initial review and have made appropriate revisions throughout the manuscript. The objectives, methods, results, and discussion are now presented more clearly, and the manuscript has been strengthened considerably.

I have no further major comments and believe the manuscript is suitable for publication.

Response: We sincerely thank Reviewer #4 for the careful reassessment and for confirming that the manuscript has been strengthened and is suitable for publication.

Additional editorial checks

The manuscript was additionally reviewed for consistency of terminology, table headings, methodological reporting, and alignment between participant findings and recommendations. A clean revised manuscript and a marked-up manuscript with tracked changes are submitted separately, as requested.

We thank the Editor and Reviewers again for their constructive guidance and consideration of our revised manuscript.

Sincerely,

Dr. Anil Fastenau, on behalf of all authors

Senior Technical Advisor

Marie Adelaide Leprosy Centre, Karachi, Pakistan
