# Supplementary material for: Delayed diagnosis as a barrier to Zero Leprosy: Lived experiences from Pakistan
Source: PLOS Glob Public Health. 2026 Aug 27;6(8):e0007184. doi: 10.1371/journal.pgph.0007184 (PMC13521354; doi:10.1371/journal.pgph.0007184)
Supplement: Supplementary file 3 [file pgph.0007184.s001.docx]

**Interview Guide**

INTRODUCTION

- Interviewer introduces him/herself and the study.
- Interviewer gives instructions about the interview.
- Interviewer asks if participant has any questions about the interview/study.
- (Interviewer is free to discuss any other topics/small talk to create trust.)
- **Informed consent:** if written informed consent was not possible, verbal consent needs to be obtained and recorded.

| SEMI-STRUCTURED QUESTIONS | |
| --- | --- |
| Exploring knowledge about ‘Leprosy’ | - **Could you tell me a bit about your health?** - **Could you tell me about the disease you are diagnosed with?** - **Do you know what it is called?** (If the participant uses another word to describe Leprosy disease, the interviewer will use the same word to refer to Leprosy disease throughout the interview). - **How do you feel about the diagnosis?** - **What do you know about Leprosy?** |
| Exploring the route to diagnosis | 1. **In your own words, could you briefly guide me through your journey to getting diagnosed?**  - When did you notice the first symptoms? What symptoms did you notice? How did you notice the first symptoms? - What did you think? (Were you thinking about Leprosy?) What did you do? Why? - Where did you first seek advice from after observing these symptoms? What did they tell you? - When did you visit a doctor, why? What happened afterwards? How did you experience the consultation with the doctor(s)? - When did you receive your diagnosis? - How much time did pass between noticing your first symptoms and the moment of diagnosis? (How old were you when you first noticed symptoms, how old were you when you received your diagnosis?) |
| Exploring the reasons for delay in diagnosis | 2. **From previous studies, we know that some time can pass before someone receives a diagnosis. There might pass weeks, months or years between the moment that someone notices the first symptoms and the moment of obtaining the diagnosis.** For example, you told me… **This time is what we call the ‘delay’.**   **What do you think were the reasons for this delay in your diagnosis?**  **In your opinion, what can be other reasons for the delay in diagnosis?**   - What are the reasons for not immediately receiving Leprosy diagnosis after noticing the first symptoms? (e.g. knowledge, awareness…) - What are reasons related to other people (e.g your family, community, health worker…)? - What are reasons related to the setting (e.g. access to health care, health system, resources/money, stigma, materials…)?  3. **Which barriers did you experience to get Leprosy diagnosis?**  - In your opinion, which reasons can you think of that caused you to not receive Leprosy diagnosis immediately after noticing the first symptoms?  4. **Do you know other people diagnosed with Leprosy?** (if yes) **Did they experience a delay in diagnosis? Which barriers do you think or know others experience to get Leprosy diagnosis?**    - Reasons related to people (family, community, health worker)    - Reasons related to setting (access to health care, health system, resources/money, stigma, materials) 5. **Some Leprosy patients do not seek help after noticing the first symptoms.**   **What do you think about this?**   - Do you think that most leprosy patients do not seek help after noticing symptoms? Why? (if yes) What do you think are the reasons that people do not seek help immediately?  6. **Do you think that Leprosy patients need several consultations before they receive the Leprosy diagnosis?** (if yes) **Why?**  - What do you think are the reasons that patients need several consultations before receiving Leprosy diagnosis?  7. **Before moving on, are there any other reasons for delay in diagnosis that we have not discussed yet?** |
| Exploring suggestions for improvement | 8. **What helped you in getting your leprosy diagnosis?** 9. **Is there anything you would wish for during the process of getting your leprosy diagnosis? If so, what and why?** 10. **In your opinion, what do you think is necessary to receive Leprosy diagnosis more quickly? Why do you think so?** |

ENDING

- These were my questions. Is there anything else related to the topic that we have not covered yet and that you would like to discuss?
- How do you feel about the interview? Do you have any questions?
- Thank you very much for your participation. May we contact you again in case additional questions come up?
- Would you like to receive the results of the study once it is finished? (if yes) How can we reach out to you?
