# Supplementary material for: Delayed diagnosis as a barrier to Zero Leprosy: Lived experiences from Pakistan
Source: PLOS Glob Public Health. 2026 Aug 27;6(8):e0007184. doi: 10.1371/journal.pgph.0007184 (PMC13521354; doi:10.1371/journal.pgph.0007184)
Supplement: Supplementary file 4 [file pgph.0007184.s002.docx]

**Sociodemographic questionnaire and Interview feedback**

Participant:	Date:

Location:	Duration of interview:

**How old are you?**

**Where do you live? (city? rural?)**

**Do you identify as man, woman or other?**

**Do you work?** (if yes) **What kind of work do you do?**

**Did you receive any formal education?** (if yes) **Where did you receive your education from?**

**Rating of the interview:	1	2	3	4	5**

**Comments and reflections on the interview:**
