# Supplementary material for: Delayed diagnosis as a barrier to Zero Leprosy: Lived experiences from Pakistan
Source: PLOS Glob Public Health. 2026 Aug 27;6(8):e0007184. doi: 10.1371/journal.pgph.0007184 (PMC13521354; doi:10.1371/journal.pgph.0007184)
Supplement: Supplementary file 5 [file pgph.0007184.s004.docx]

**Point by Point Responses**

**Response to Reviewer #1**

We sincerely thank Reviewer #1 for the thoughtful, constructive, and encouraging evaluation of our manuscript. We are grateful for the reviewer’s recognition of the value of the lived-experience perspective and for the helpful suggestions to strengthen the methodological transparency, contextualization, and policy relevance of the paper. We have revised the manuscript accordingly and respond to each point below.

**Reviewer comment 1:**
*This manuscript presents a valuable qualitative exploration of the barriers to timely leprosy diagnosis in Pakistan, a “low-endemic” setting that continues to face high rates of Grade 2 Disability (G2D) at the time of diagnosis. By focusing on the lived experiences of those already suffering from visible deformities, the authors provide critical insights into why “Zero Leprosy” remains elusive despite the availability of free treatment. The study is well-structured, ethically sound, and directly addresses the human and systemic failures that sustain hidden transmission.*

**Response:**
We sincerely thank the reviewer for this generous and encouraging assessment of our work. We are grateful that the reviewer recognized the importance of centering the lived experiences of persons affected by leprosy with visible disability, as this was one of the main aims of the study. We also appreciate the reviewer’s emphasis on the relevance of these findings for understanding hidden transmission and the ongoing challenges to achieving Zero Leprosy in Pakistan.

**Reviewer comment 2:**
*Human-Centric Perspective: Unlike many quantitative studies that focus solely on “delay intervals,” this research prioritizes the narratives of affected individuals. This approach highlights the “human cost” of late detection and reveals gaps that surveillance data alone cannot capture.*

**Response:**
We thank the reviewer for highlighting this strength. We agree that the lived-experience perspective adds an important dimension to existing quantitative and programmatic evidence on diagnostic delay. We have retained this focus throughout the manuscript and further emphasized the value of participant narratives in the Discussion.

**Location of revision:**
Discussion section, subsection “Strengths of lived-experience perspectives.”

**Reviewer comment 3:**
*The analysis successfully categorizes barriers into individual, community, and health system levels. This holistic view is essential for programmatic planning, as it shows that clinical expertise alone is insufficient if social stigma or financial “opportunity costs” prevent patients from reaching the clinic.*

**Response:**
We thank the reviewer for this positive assessment. We agree that diagnostic delay cannot be addressed through clinical training alone and that social, financial, and structural barriers must be considered together. This was one of the key reasons for organizing the Results around individual-, community-, and health system-level barriers.

**Location of revision:**
Discussion section and Recommendations section.

**Reviewer comment 4:**
*A significant strength is the inclusion of a dedicated section for participant-suggested strategies. The recommendation to utilize treated patients as “agents of change” and peer educators is a culturally grounded and empowering solution for reducing stigma and encouraging early care-seeking.*

**Response:**
We are grateful for this comment and are pleased that the reviewer recognized the value of including participant-suggested strategies. We agree that persons affected by leprosy are not only study participants but also important knowledge holders and potential agents of change in Zero Leprosy efforts.

**Location of revision:**
Results section, subsection “Involvement of persons affected by leprosy,” and Recommendations section, subsection “Actively involve persons affected by leprosy.”

**Reviewer comment 5:**
*The use of thematic content analysis with ATLAS.ti 24 and the explicit mention of reaching data saturation at 19 interviews lend credibility to the qualitative findings.*

**Response:**
We thank the reviewer for acknowledging this methodological strength. In response to the reviewer’s later methodological comments, we revised the Methods section to provide greater clarity on transcription, translation, quote handling, and analysis while retaining the reference to thematic content analysis supported by ATLAS.ti 24.

**Location of revision:**
Methodology section, subsection “Data Collection and Analysis.”

**Reviewer comment 6:**
*The authors followed the Declaration of Helsinki and obtained approval from the Marie Adelaide Leprosy Centre (MALC) Ethical Review Committee. The use of thumbprints for participants with disabilities or limited literacy ensures inclusive ethical practice.*

**Response:**
We thank the reviewer for recognizing the ethical considerations taken in this study. Ensuring inclusive and respectful consent procedures was particularly important given the vulnerability of some participants and the stigma associated with leprosy.

**Reviewer comment 7:**
*The authors note that non-simultaneous translation was used, meaning statements were “summarized rather than literally reproduced”. In qualitative research, the exact phrasing often carries significant weight. While the authors took steps to minimize errors, this remains a limitation that could have impacted the “verbatim” nature of the transcriptions mentioned later. Recommendation: Discuss more explicitly how summarized translation might have affected the “richness” of the data and whether any back-translation or member-checking was used to ensure accuracy.*

**Response:**
We thank the reviewer for this important and very helpful comment. We agree that our previous wording could have created ambiguity, particularly because we referred to “verbatim” transcription while also noting that some Urdu responses were summarized through non-simultaneous interpretation. We have therefore revised the Methods section to distinguish more clearly between verbatim transcription of available English dialogue and interpreter-mediated translated participant accounts. We also now explicitly state that formal back-translation and participant member-checking were not conducted.

In addition, we expanded the Limitations section to acknowledge that non-simultaneous interpretation may have reduced the linguistic richness of the data, including idiomatic expressions, emotional emphasis, and culturally specific phrasing.

**Location of revision:**
Methodology section, subsection “Data Collection and Analysis,” and Discussion section, subsection “Limitations.”

**Reviewer comment 8:**
*The sample consists of 14 males and only 5 females. While the manuscript discusses gender-specific barriers (e.g., the need for male chaperones and lack of female healthcare workers), the small number of female participants may limit the depth of these findings. Recommendation: Acknowledge this skew more clearly as a limitation and suggest that future research specifically target female populations to fully understand the intersection of gender and disability in this context.*

**Response:**
We thank the reviewer for this important observation. We agree that the male-skewed sample limits the depth and transferability of our findings on gender-specific barriers. Although the gender-related findings are valuable, we recognize that they should be interpreted cautiously given the limited number of female participants.

**Location of revision:**
Discussion section, subsection “Limitations,” and Recommendations section, subsection “Enhance gender-sensitive and inclusive service delivery.”

**Reviewer comment 9:**
*Some participants reported delays of up to 30 years. While the authors mention recall bias, they should further discuss how the health system context in Pakistan 30 years ago differs from today. This would help clarify if the reported barriers are historical or persistent in the current landscape.*

**Response:**
We thank the reviewer for this thoughtful suggestion. We agree that very long diagnostic delays require careful interpretation because some participant experiences may reflect historical health system conditions that differ from the current context. At the same time, several barriers described by participants with long delays were also reported by more recently diagnosed participants, suggesting that some challenges remain persistent.

**Location of revision:**
Discussion section, subsection “Limitations.”

**Reviewer comment 10:**
*The current statement says data is not available due to “sensitive information”. While participant privacy is paramount in leprosy research, PLOS policies usually require the sharing of anonymized/de-identified data. Recommendation: Provide a more detailed justification for why de-identified transcripts cannot be made available in a repository, or clarify if a “minimal data set” can be generated.*

**Response:**
We thank the reviewer for raising this important point. We agree that the original Data Availability Statement was too brief and did not sufficiently explain why full de-identified transcripts cannot be made publicly available. Given the small sample size, the stigmatized nature of leprosy, and the detailed personal information contained in the interviews, we believe that full de-identification cannot reliably prevent deductive identification. However, we recognize the importance of transparency and have therefore clarified that a de-identified minimal dataset may be made available upon reasonable request and subject to ethics approval.

**Location of revision:**
Declarations section, subsection “Availability of data and materials.”

**Reviewer comment 11:**
*The manuscript frequently references “Zero Leprosy”. The Discussion could be strengthened by more explicitly linking the findings to the three pillars of the Global Leprosy Strategy: zero transmission, zero disability, and zero discrimination. For example, highlight how “label-based stigma” specifically impedes the “zero discrimination” goal.*

**Response:**
We thank the reviewer for this excellent suggestion. We agree that the Discussion would benefit from a clearer and more explicit link between our findings and the three pillars of Zero Leprosy. We have therefore revised the Discussion to show how delayed diagnosis undermines zero transmission, zero disability, and zero discrimination.

**Location of revision:**
Discussion section, subsection “Implications for Zero Leprosy in Pakistan.”

**Reviewer comment 12:**
*The paper notes that leprosy services are largely NGO-driven in Pakistan. The authors should expand on the policy implications of this. If the goal is “Zero Leprosy” by 2030, how can the government transition from a “passive” observer to a leader in integrating leprosy into the national primary healthcare system?*

**Response:**
We thank the reviewer for this very relevant and constructive recommendation. We agree that the NGO-driven structure of leprosy services in Pakistan has important policy implications. While NGOs such as MALC have played an essential role in maintaining specialized expertise and care, achieving Zero Leprosy requires stronger government ownership, integration into primary healthcare, and sustainable financing.

**Location of revision:**
Discussion section, subsection “Implications for Zero Leprosy in Pakistan.”

**Reviewer comment 13:**
*The manuscript mentions Pakistan is classified as “low endemic”. It would be helpful to briefly provide the WHO threshold for this classification in the Introduction to orient readers unfamiliar with leprosy epidemiology.*

**Response:**
We thank the reviewer for this helpful suggestion. We agree that readers who are less familiar with leprosy epidemiology would benefit from a brief explanation of the WHO threshold for elimination as a public health problem.

**Location of revision:**
Introduction section, paragraph discussing Pakistan’s classification as a low-endemic country.

**Reviewer comment 14:**
*While generally well-written, ensure that quotes rewritten to the “first person” (as mentioned in the Methods) do not inadvertently change the participant’s original meaning or emphasis.*

**Response:**
We thank the reviewer for this careful observation. We agree that quote handling must be transparent, especially in interpreter-mediated qualitative research. We have therefore revised the Methods section to clarify that quotes were only lightly edited for readability and that conversion into first person was done only when meaning and emphasis were preserved.

**Location of revision:**
Methodology section, subsection **“**Data Collection and Analysis.”

**Reviewer comment 15:**
*The authors correctly identify the absence of children in the study population as a limitation. Given that child leprosy cases are a primary indicator of recent transmission, emphasize this more strongly in the “Recommendations” for future research.*

**Response:**
We thank the reviewer for this important suggestion. We agree that child leprosy is a key indicator of recent transmission and that the exclusion of children deserves stronger emphasis, especially in the context of Zero Leprosy and transmission interruption.

**Location of revision:**
Recommendations section, subsection “Link early diagnosis to prevention and transmission interruption.”

__________________________________________________________________________

**Response to Reviewer #2**

We sincerely thank Reviewer #2 for the thoughtful, encouraging, and constructive evaluation of our manuscript. We are grateful for the reviewer’s recognition of the relevance of the study, the appropriateness of the qualitative design, the ethical conduct of the research, and the contribution of lived-experience perspectives to the Zero Leprosy agenda. We have made minor revisions to improve conciseness, fluency, and the programmatic relevance of the manuscript, and we respond to each point below.

**Reviewer comment 1:**
*The manuscript makes a relevant and timely contribution to the field of global public health by exploring in depth the delay in leprosy diagnosis in Pakistan from the perspective of people diagnosed with grade 2 disability. The study is well aligned with the scope of PLOS Global Public Health and the principles of the Zero Leprosy agenda, highlighting the clinical, programmatic, social, and ethical implications of late diagnosis in low-endemicity contexts.*

**Response:**
We sincerely thank the reviewer for this generous and encouraging assessment. We are grateful that the reviewer recognized the relevance of the study for global public health and its alignment with the Zero Leprosy agenda. We agree that diagnostic delay in low-endemic settings has clinical, programmatic, social, and ethical implications, and we have further strengthened this connection in the revised Discussion.

**Location of revision:**
Discussion section, especially the subsections “Delay, disability, and the paradox of ‘low endemic’ settings” and “Implications for Zero Leprosy in Pakistan.”

**Reviewer comment 2:**
*From a methodological point of view, the qualitative design is appropriate for the proposed objectives and was conducted rigorously. The eligibility criteria, the recruitment process, data collection through in-depth interviews, and thematic analysis are clearly described. The use of dedicated software for qualitative analysis, the combination of deductive and inductive coding, and the explicit statement of the saturation point lend transparency and credibility to the analytical process. The limitations of the study—including possible recall bias and challenges related to translation—are recognized and discussed adequately, which reinforces the ethical and reflective stance of the authors.*

**Response:**
We thank the reviewer for this positive assessment of the study design and methodological reporting. We are pleased that the reviewer found the qualitative approach, recruitment strategy, data collection, analytical process, and discussion of limitations to be appropriate and transparent. In response to Reviewer #1 and to further improve clarity, we revised the Methods and Limitations sections to more precisely describe interpreter-mediated translation, transcription, quote handling, and the absence of formal back-translation or member-checking.

**Location of revision:**
Methodology section, subsection “Data Collection and Analysis,” and Discussion section, subsection “Limitations.”

**Reviewer comment 3:**
*The conclusions presented are coherent and supported by the data, respecting the interpretative limits of a qualitative study. The findings are well anchored in the existing literature and broaden the understanding of how individual, community, and structural factors interact to prolong the time to diagnosis, resulting in preventable disability and perpetuation of transmission. The emphasis on lived experiences adds substantial value to the debate, complementing quantitative studies and analyses focused exclusively on the health system.*

**Response:**
We sincerely thank the reviewer for this supportive comment. We agree that lived-experience perspectives are essential for understanding how individual, community, and health system barriers interact in real diagnostic pathways. We have retained this emphasis and slightly strengthened the manuscript’s discussion of how qualitative findings can complement quantitative and health-system-focused evidence.

**Location of revision:**
Discussion section, subsection “Strengths of lived-experience perspectives,” and Recommendations section, subsection “Link early diagnosis to prevention and transmission interruption.”

**Reviewer comment 4:**
*Regarding data availability, although the primary data are not accessible in a public repository, the justification presented—involving the sensitivity of the information and the protection of a vulnerable group—is adequate and consistent with the PLOS data policy for qualitative research. The clear indication of an institutional contact point for data requests, coupled with the presentation of representative excerpts from the interviews, allows for the assessment of the robustness of the analyses and the coherence between data and conclusions.*

**Response:**
We thank the reviewer for recognizing the importance of protecting participants while maintaining transparency. We are grateful that the reviewer considered the revised Data Availability Statement appropriate for sensitive qualitative data involving a vulnerable and potentially identifiable population. We retained the controlled-access approach and the institutional contact point for data requests.

**Location of revision:**
Declarations section, subsection “Availability of data and materials.”

**Reviewer comment 5:**
*From an ethical standpoint, the study demonstrates full compliance with international standards, with approval by an ethics committee, obtaining informed consent, and appropriate confidentiality measures. There are no indications of concerns related to research ethics, publication ethics, or duplicate publication.*

**Response:**
We thank the reviewer for this positive assessment. Ethical conduct, confidentiality, and inclusive informed consent procedures were central to the study, particularly given the stigma and vulnerability associated with leprosy and visible disability.

**Reviewer comment 6:**
*Regarding the manuscript's presentation, the text is clear, well-structured, and written in appropriate standard English. However, a light editorial review is recommended to improve conciseness and fluency in some sections, especially in the Results, which are at times lengthy and could be summarized without analytical loss. Minor language adjustments and reduction of repetitions can further increase the text's accessibility to non-specialist readers.*

**Response:**
We thank the reviewer for this helpful suggestion. We agree that some parts of the Results section could be made more concise without losing analytical depth or the participant-centred character of the manuscript. We therefore conducted a light editorial review and shortened selected passages in the Results to reduce repetition, improve fluency, and enhance accessibility for non-specialist readers while retaining representative participant quotations.

**Location of revision:**
Results section, particularly the subsections “Limited healthcare-seeking behavior,” “Limited leprosy expertise among healthcare workers,” and “Awareness campaigns.” Minor language edits were also made in the Abstract, Methodology, Results, and Limitations sections.

**Reviewer comment 7:**
*As a constructive suggestion, the authors could consider: reinforcing, in a more concise way in the Discussion, the link between diagnostic delay and concrete programmatic implications for health systems in low-endemicity countries.*

**Response:**
We thank the reviewer for this valuable suggestion. We agree that the Discussion should make the programmatic implications for low-endemic settings more explicit. We therefore added a concise paragraph highlighting concrete priorities for health systems in low-endemic countries, including maintaining leprosy competence within general health services, embedding sensory testing and skin examination into frontline and dermatology care, establishing clear referral pathways, and using G2D at diagnosis as an indicator of diagnostic timeliness.

**Location of revision:**
Discussion section, subsection “Delay, disability, and the paradox of ‘low endemic’ settings.”

**Reviewer comment 8:**
*As a constructive suggestion, the authors could consider: indicating more explicitly how the qualitative findings can inform future quantitative research or mixed studies, especially regarding the interruption of transmission.*

**Response:**
We thank the reviewer for this excellent recommendation. We agree that the qualitative findings can guide future quantitative and mixed-methods studies by identifying delay factors that should be measured systematically. We therefore added a recommendation that future studies assess the relative contribution of factors such as symptom recognition, first provider contact, referral failures, gender-related access barriers, stigma, and costs. We also added that prospective studies could link diagnostic delay intervals with G2D, household contact screening outcomes, SDR-PEP eligibility, and child case detection to better understand the relationship between diagnostic delay and transmission interruption.

**Location of revision:**
Recommendations section, subsection “Link early diagnosis to prevention and transmission interruption.”

__________________________________________________________________________

**Response to Reviewer #3**

We sincerely thank Reviewer #3 for the thoughtful, detailed, and constructive evaluation of our manuscript. We are grateful for the reviewer’s recognition of the relevance of the study for neglected tropical diseases, health systems strengthening, stigma, health-seeking behaviour, and disease elimination strategies. We particularly appreciate the reviewer’s suggestions to strengthen the interpretive depth of the analysis, clarify the implications of our sampling strategy, and link the recommendations more explicitly to the qualitative findings.

**Reviewer comment 1:**
*This manuscript presents a qualitative investigation of diagnostic delay in leprosy in Pakistan, drawing on lived experiences of individuals with grade 2 disability (G2D). The study provides rich insights into multi-level barriers (individual, community, and health system) contributing to delayed diagnosis and ongoing transmission.*

*The manuscript is highly relevant to global public health, particularly within the domains of: Neglected tropical diseases (NTDs), Health systems strengthening, Stigma and health-seeking behavior, Disease elimination strategies.*

*The study is well-structured, analytically coherent, and policy-relevant, and makes a meaningful contribution to the “Zero Leprosy” discourse.*

**Response:**
We sincerely thank the reviewer for this generous and encouraging assessment. We are grateful that the reviewer recognized the relevance of our study for global public health, health systems strengthening, stigma, health-seeking behaviour, and disease elimination. We also appreciate the reviewer’s acknowledgement that the manuscript contributes meaningfully to the Zero Leprosy discourse. We have revised the manuscript to further strengthen the analytical interpretation and policy relevance of the findings.

**Location of revision:**
Discussion section, particularly the subsections “Stigma, non-disclosure, and hidden transmission,” “Gender, marginalization, and delayed interruption of transmission,” and “Implications for Zero Leprosy in Pakistan.”

**Reviewer comment 2:**
*The manuscript makes several important contributions: positions diagnostic delay as a central driver of transmission, not just disability; integrates lived-experience perspectives, often underrepresented in programmatic research; highlights the paradox of low-endemic settings with high disability burden; provides actionable recommendations aligned with elimination strategies. The framing of diagnostic delay as a “systemic and cumulative failure” rather than a single-point issue is particularly compelling.*

**Response:**
We thank the reviewer for highlighting these contributions. We agree that diagnostic delay should be understood not only as a cause of disability, but also as a driver of ongoing transmission and as a reflection of cumulative failures across social, clinical, and health system levels. We have retained this framing and strengthened the Discussion to further clarify what these findings mean structurally and programmatically.

**Location of revision:**
Discussion section, especially “Diagnostic delay as a driver of ongoing transmission,” “Delay, disability, and the paradox of ‘low endemic’ settings,” and “Implications for Zero Leprosy in Pakistan.”

**Reviewer comment 3:**
*The manuscript successfully links diagnostic delays to transmission dynamics, missed prevention opportunities, health system gaps, stigma and concealment. For example, the discussion clearly articulates that prolonged undiagnosed periods create repeated opportunities for transmission. This is a strong and policy-relevant argument. Strength noted: The integration of Zero Leprosy framework is appropriate and well-developed.*

**Response:**
We sincerely thank the reviewer for this positive assessment. We are pleased that the reviewer found the conceptual framing and integration of the Zero Leprosy framework appropriate and policy-relevant. In the revised manuscript, we retained this framing and further strengthened the link between diagnostic delay, health system gaps, stigma, and the three pillars of Zero Leprosy: zero transmission, zero disability, and zero discrimination.

**Location of revision:**
Discussion section, subsections “Diagnostic delay as a driver of ongoing transmission,” “Missed opportunities for prevention and post-exposure prophylaxis,” and “Implications for Zero Leprosy in Pakistan.”

**Reviewer comment 4:**
*The thematic analysis is generally robust, supported by participant quotations. However: At times, the manuscript leans toward descriptive reporting rather than deeper analytical interpretation. Some themes (e.g., stigma, gender barriers) could be more explicitly linked to existing theoretical frameworks (e.g., stigma theory, structural violence). Recommendation: Strengthen interpretive depth by engaging with theory and moving beyond “what participants said” to “what this means structurally”.*

**Response:**
We thank the reviewer for this very helpful recommendation. We agree that the manuscript could further strengthen its interpretive depth by linking the findings to broader conceptual frameworks. We therefore expanded the Discussion to interpret leprosy-related stigma not only as an interpersonal experience, but also as anticipatory and internalized stigma that can shape disclosure and care-seeking behaviour. We also added a structural interpretation of diagnostic delay, showing how poverty, gender norms, unstable housing, dependence on daily wages, and uneven access to competent healthcare constrain individual choices and make late diagnosis more likely.

**Location of revision:**
Discussion section, subsections “Stigma, non-disclosure, and hidden transmission” and “Gender, marginalization, and delayed interruption of transmission.”

**Reviewer comment 5:**
*The study focuses exclusively on individuals with grade 2 disability, which is both a strength and a limitation. Strength: captures severe consequences of delay. Limitation: excludes early-stage patients and caregivers. The manuscript acknowledges this limitation, but it should more clearly discuss how this focus may bias findings toward extreme delay narratives.*

**Response:**
We thank the reviewer for this important observation. We agree that focusing exclusively on persons with G2D is both a strength and a limitation. This focus allowed us to understand the severe consequences of diagnostic delay, but it may also overrepresent more prolonged and severe diagnostic pathways. We have therefore expanded the Limitations section to make clear that persons diagnosed earlier, before visible disability developed, may have experienced different barriers, shorter pathways, or more effective referral processes.

**Location of revision:**
Discussion section, subsection “Limitations.”

**Reviewer comment 6:**
*Participants reported delays ranging from months to decades. Given the retrospective nature: recall bias is acknowledged but could be further emphasized. Consider discussing how memory reconstruction may influence delay estimates.*

**Response:**
We thank the reviewer for this valuable suggestion. We agree that reported delay durations should be interpreted cautiously, especially when participants described diagnostic pathways that extended over many years or decades. We have therefore expanded the Limitations section to emphasize that symptom onset, first consultations, referrals, and diagnosis dates may be difficult to recall precisely. We also added that delay estimates may be influenced by memory reconstruction, where earlier symptoms or care-seeking events are interpreted through the lens of a later diagnosis and disability.

**Location of revision:**
Discussion section, subsection “Limitations.”

**Reviewer comment 7:**
*The manuscript effectively identifies misdiagnosis, lack of clinical awareness, and weak referral systems. However, the critique could be sharpened by distinguishing between primary care failures, specialist-level gaps, and structural vs operational issues.*

**Response:**
We thank the reviewer for this constructive recommendation. We agree that distinguishing the different levels of health system barriers makes the programmatic implications clearer. We have therefore added a paragraph in the Discussion that differentiates between primary care-level gaps, specialist-level gaps, operational barriers, and structural barriers. This distinction helps clarify that different types of interventions are needed, including clinical training, referral tools, patient navigation, and stronger government stewardship.

**Location of revision:**
Discussion section, subsection “Delay, disability, and the paradox of ‘low endemic’ settings.”

**Reviewer comment 8:**
*The recommendations are comprehensive and well-aligned with findings, including awareness campaigns, patient involvement, training healthcare workers, strengthening referral pathways, and gender-sensitive interventions. However, some recommendations appear broad and programmatic rather than directly derived from qualitative insights. Recommendation: Explicitly link each recommendation to specific findings/themes.*

**Response:**
We thank the reviewer for this helpful suggestion. We agree that the link between the recommendations and the qualitative findings should be made more explicit. We therefore revised the opening paragraph of the Recommendations section to directly map each recommendation onto the main themes identified in the analysis, including limited symptom recognition, uncertainty about where to seek care, repeated misdiagnosis, weak referral experiences, stigma and non-disclosure, and gendered and structural barriers.

**Location of revision:**
Recommendations section, opening paragraph.

**Reviewer comment 9:**
*Replace “patients” with: “persons affected by leprosy” (person-centered language).*

**Response:**
We thank the reviewer for this important suggestion. We agree that person-centred language is essential in leprosy research, particularly given the history of stigma and discrimination associated with the disease. We reviewed the manuscript and replaced “patients” with “persons affected by leprosy,” “participants,” or “individuals affected by leprosy” where appropriate. In a few clinical or quoted contexts, terminology was retained only where needed for clarity or where it appeared in participant quotations.

**Location of revision:**
Throughout the manuscript, especially the Results, Discussion, and Recommendations sections.

**Reviewer comment 10:**
*Quotations are appropriate and illustrative. Consider slightly reducing repetition and prioritizing the most analytically rich excerpts.*

**Response:**
We thank the reviewer for this helpful comment. We agree that the quotations should remain illustrative while avoiding repetition. We therefore conducted a light editorial review of the Results section, reduced repetitive explanatory text, and retained quotations that most clearly support the analytical themes. We also removed duplicated text that remained from tracked revisions.

**Location of revision:**
Results section, particularly the subsections “Limited healthcare-seeking behavior,” “Limited leprosy expertise among healthcare workers,” and “Awareness campaigns.”

**Reviewer comment 11:**
*The manuscript highlights gender barriers (e.g., limited access to female healthcare workers). This is important but could be expanded: Are delays systematically longer for women? How do gender norms shape concealment or care-seeking?*

**Response:**
We thank the reviewer for this important and thoughtful comment. We agree that gender norms may shape diagnostic delay through restrictions on independent mobility, dependence on male relatives for transport or permission, and discomfort with examination by male healthcare workers. At the same time, our sample included only five female participants, so we cannot conclude whether delays were systematically longer among women than men. We have therefore expanded the Discussion to interpret the gender-related findings cautiously and to identify this as an important area for future research.

**Location of revision:**
Discussion section, subsection “Gender, marginalization, and delayed interruption of transmission,” and Discussion section, subsection “Limitations.”

**Reviewer comment 12:**
*Ethical approval and consent are clearly stated. Data availability is restricted due to sensitivity, which is appropriate.*

**Response:**
We thank the reviewer for recognizing the ethical safeguards and the appropriateness of the restricted data availability approach. Given the sensitive and potentially identifiable nature of the qualitative data, especially in a small and stigmatized population, we have retained the controlled-access data availability statement.

**Location of revision:**
Declarations section, subsection “Availability of data and materials.”

**Reviewer comment 13:**
*Overall well-written. Minor grammatical edits may improve flow in some sections.*

**Response:**
We thank the reviewer for this helpful suggestion. We conducted a light language and style review to improve clarity, conciseness, and flow. We also removed duplicated text that remained from tracked revisions and harmonized terminology throughout the manuscript.

**Location of revision:**
Minor edits throughout the manuscript, particularly in the Abstract, Methodology, Results, Discussion, Recommendations, and Declarations sections.

__________________________________________________________________________

**Response to Reviewer #4**

We sincerely thank Reviewer #4 for the detailed, constructive, and thoughtful evaluation of our manuscript. We are grateful for the reviewer’s recognition of the public health relevance of diagnostic delay in leprosy, the value of lived-experience perspectives, and the importance of the study within the Zero Leprosy framework. We have carefully revised the manuscript to strengthen methodological transparency, conceptual clarity, internal coherence, and alignment across the objective, methods, results, discussion, and recommendations.

**Reviewer comment 1:**
*This manuscript addresses a highly relevant public health issue, diagnostic delay in leprosy, and its implications for disability and ongoing transmission in Pakistan. The focus on individuals with grade 2 disability (G2D) provides important insight into late-stage diagnosis and contributes valuable patient-centered perspectives to the literature.*

*The qualitative approach is appropriate for exploring lived experiences, and the manuscript is generally well-written and conceptually grounded within the “Zero Leprosy” framework.*

*However, several methodological, analytical, and reporting issues require clarification and strengthening to enhance the scientific rigor, internal coherence, and interpretability of the findings.*

**Response:**
We sincerely thank the reviewer for this encouraging and constructive overall assessment. We appreciate the recognition of the manuscript’s relevance, qualitative approach, and contribution to patient-centred evidence on late-stage leprosy diagnosis. We have revised the manuscript substantially to improve methodological detail, clarify the conceptual framework, strengthen reflexivity, and ensure closer alignment between the study objective, methods, findings, discussion, and recommendations.

**Reviewer comment 2:**
*The stated objective focuses on exploring reasons for delayed diagnosis. However, the methods and results indicate that the study also captured participants’ suggestions for improving services. This broader scope is not reflected in the objective.*

*The authors should revise the objective to clearly reflect all components of the study, including both: reasons for diagnostic delay, and proposed solutions or recommendations from participants. Ensuring alignment across the objective, methods, results, and discussion will improve the overall coherence of the manuscript.*

**Response:**
We thank the reviewer for this important observation. We agree that the original objective did not fully reflect the scope of the study, as the interviews explored not only reasons for diagnostic delay but also participant-suggested strategies to reduce delay. We have therefore revised the objective to explicitly include both components. This revision improves alignment across the Abstract, Introduction, Methods, Results, Discussion, and Recommendations.

**Location of revision:**
Abstract, Background section; Introduction, final paragraph; Methodology section, subsection “Data Collection and Analysis.”

**Reviewer comment 3:**
*The process for identifying and recruiting study participants is insufficiently described. The manuscript notes that Pakistan reports approximately 250 new leprosy cases annually, with a significant proportion presenting with Grade 2 disability. However, it is unclear how participants were sampled from this population, what inclusion criteria were applied beyond disability grade, and how participants were approached and invited.*

*Additionally, the manuscript states that the year of diagnosis was not a restriction, implying inclusion of individuals diagnosed many years prior. It is unclear how such participants were identified and whether registries or facility records were used.*

*Recommendation: The authors should clearly describe sampling strategy and recruitment process, explain how older cases were traced, justify inclusion of long-term retrospective participants, and clarify whether sampling aimed at saturation across key characteristics.*

**Response:**
We thank the reviewer for this helpful recommendation. We agree that the recruitment process required greater methodological detail. We have revised the Methodology section to clarify that participants were purposively identified through MALC facility records, MALC headquarters, selected subcentres, and follow-up networks. We now explain that older cases were identifiable because many persons affected by leprosy remain connected to MALC through disability care, rehabilitation, ulcer care, follow-up, or social support services after completion of MDT. We also expanded the inclusion and exclusion criteria and clarified that sampling aimed to capture variation in gender, age, residence, educational background, employment status, and time since diagnosis, while maintaining the focus on persons with G2D.

**Location of revision:**
Methodology section, subsection “Study Setting and Study Population”

**Reviewer comment 4:**
*Participants reported delays up to 30 years and diagnoses spanning decades. This introduces recall bias and contextual inconsistency, as the health system in 1989 vs 2024 is not comparable. Currently, this is acknowledged but not adequately addressed analytically.*

*Recommendation: Stratify or reflect on differences between recent vs older diagnoses, discuss how changing health systems may influence findings, and consider limiting interpretation or clearly framing findings as cross-generational narratives.*

**Response:**
We thank the reviewer for this important point. We agree that the inclusion of participants diagnosed across several decades introduces temporal heterogeneity and requires careful interpretation. We have therefore revised the Results and Limitations to frame the findings as cross-generational narratives of diagnostic delay rather than as a direct assessment of a single, uniform health system context. We also clarified that some barriers may have been shaped by the historical context of diagnosis, while others appear persistent across time, particularly limited awareness, stigma, weak referral pathways, and insufficient leprosy expertise.

**Location of revision:**
Results section, opening paragraph; Discussion section, subsection “Limitations.”

**Reviewer comment 5:**
*There is no discussion of the researchers' positionality, which is expected in qualitative studies. Missing elements include: who conducted interviews, insider vs outsider status, relationship to MALC, and power dynamics between the interviewer and participants.*

*Recommendation: Include a reflexivity statement, addressing researcher background and potential influence on data collection and interpretation.*

**Response:**
We thank the reviewer for raising this important methodological issue. We agree that reflexivity is essential in qualitative research, particularly when research is conducted in collaboration with service-providing institutions. We have added a reflexivity statement describing the backgrounds of the research team, the role of MALC/GLRA staff in supporting interviews, the benefits of insider knowledge, and the potential influence of institutional affiliation on participant responses. We also explain how confidentiality, voluntary participation, and team-based reflection during analysis were used to reduce these risks.

**Location of revision:**
Methodology section, new subsection “Reflexivity.”

**Reviewer comment 6:**
*There appears to be an inconsistency in how themes are presented: The methods mention five broad themes, including “characteristics of the disease.” In the results, this appears embedded under “individual barriers.” This weakens analytical clarity.*

*Recommendation: Present a clear and consistent thematic framework. Consider adding a figure/table showing themes and subthemes.*

**Response:**
We thank the reviewer for this helpful suggestion. We agree that the thematic structure needed clearer explanation. We have revised the Methods and Results to clarify that “characteristics of the disease” was treated as an individual-level barrier in the Results because participants described how painless, slowly progressive, and initially ambiguous symptoms influenced recognition of illness and timing of care-seeking. We also added a thematic framework table summarizing the analytical levels, main themes, and subthemes to improve clarity and consistency.

**Location of revision:**
Methodology section, subsection “Data Collection and Analysis,” and newly added Table 2: Thematic framework of barriers and participant-suggested strategies.

**Reviewer comment 7:**
*The manuscript states: “All participants experienced diagnostic delay.” However, there is no provision of an operational definition of diagnostic delay. This weakens conceptual clarity; the author could define diagnostic delay explicitly, e.g., symptom onset to diagnosis, and explain how it was estimated or avoid presenting delay values if not systematically measured.*

**Response:**
We thank the reviewer for this important clarification. We have now added an operational definition of diagnostic delay. In this study, diagnostic delay was defined as the period between the participant’s first recalled sign or symptom compatible with leprosy and the point at which a leprosy diagnosis was received from a healthcare provider. We also clarified that delay duration was estimated from participant self-report and, where available, checked against clinical or registration records. Because timing was sometimes difficult to recall precisely, we now state that delay estimates were treated as approximate and used primarily to contextualize narratives rather than as systematically measured epidemiological outcomes.

**Location of revision:**
Methodology section, subsection “Data Collection and Analysis,” and Results section, opening paragraph.

**Reviewer comment 8:**
*Some quotes under the individual thematic area use “they” instead of “I”. This creates ambiguity: Were the participants sharing lived experiences or general community perceptions? The author could clarify the distinction between personal experience vs perceived norms and ensure consistency in interpretation.*

**Response:**
We thank the reviewer for this careful observation. We agree that some participants shifted between personal accounts and broader observations about community norms. We have clarified in the Methods that, during analysis, we distinguished between participants’ personal diagnostic experiences and their broader perceptions of community or health system practices. Statements using “people” or “they” were interpreted as perceived norms or collective observations unless explicitly connected to the participant’s own experience. We also revised introductory wording around selected quotes to make this distinction clearer.

**Location of revision:**
Methodology section, subsection “Data Collection and Analysis,” and Results section, especially subsections “Individual-level barriers,” “Limited healthcare-seeking behavior,” and “Limited knowledge and awareness.”

**Reviewer comment 9:**
*The manuscript highlights gender barriers but relies heavily on female voices and does not mention how this compares with male participants.*

**Response:**
We thank the reviewer for this important comment. We agree that the gender analysis needed to be more carefully contextualized. We have revised the Discussion to clarify that the sample size does not allow conclusions about whether diagnostic delays were systematically longer among women than men. We also now note that some male participants reported long delays, particularly where symptoms were painless, livelihood pressures were high, or trust in healthcare was low. At the same time, female participants described additional gendered mechanisms, including restrictions on independent mobility, dependence on male relatives for transport or permission, and discomfort with examination by male healthcare workers. We have framed these findings cautiously and identified the need for future studies with larger female samples.

**Location of revision:**
Discussion section, subsection “Gender, marginalization, and delayed interruption of transmission,” and Discussion section, subsection “Limitations.”

**Reviewer comment 10:**
*The Discussion is strong but somewhat overextended. It introduces transmission dynamics, policy implications, and Zero Leprosy framing, yet some of these are not directly grounded in the presented data.*

*Recommendation: The author should strengthen the linkage between findings and interpretation. In addition, he should avoid introducing concepts not clearly supported by results.*

**Response:**
We thank the reviewer for this thoughtful critique. We agree that the Discussion should clearly distinguish between findings derived directly from participant narratives and broader interpretations informed by the leprosy literature. We have therefore revised the Discussion to clarify that this qualitative study did not measure transmission directly. Instead, participants’ accounts of prolonged undiagnosed periods, delayed referral, and lack of awareness are interpreted in light of existing evidence on leprosy transmission and disability. We also softened causal language where appropriate, for example by referring to prolonged undiagnosed periods as potentially creating opportunities for transmission rather than directly demonstrating transmission.

**Location of revision:**
Discussion section, subsection “Diagnostic delay as a driver of ongoing transmission,” and subsection “Implications for Zero Leprosy in Pakistan.”

**Reviewer comment 11:**
*The manuscript does not provide sufficient information about the MALC, presumably a leprosy center. The authors should clarify the role of MALC within the health system, whether it is a referral center or primary diagnostic facility, and whether all leprosy patients in the region are managed there. This context is essential for understanding the representativeness of the study population.*

**Response:**
We thank the reviewer for this important suggestion. We have expanded the Study Setting section to describe MALC’s role as one of the main non-governmental providers of leprosy diagnosis, treatment, disability care, rehabilitation, and follow-up services in Pakistan, and as a major referral centre in Karachi and surrounding areas. We also clarified that persons with suspected or confirmed leprosy may reach MALC directly, through referral from public or private healthcare providers, or through subcentres and outreach networks. Finally, we note that not all persons affected by leprosy in Pakistan are necessarily managed by MALC, meaning that the study population reflects individuals connected to MALC services rather than all persons affected by leprosy nationally.

**Location of revision:**
Methodology section, subsection “Study Setting and Study Population.”

**Reviewer comment 12:**
*Ensure all quotes align clearly with the themes they illustrate.*

**Response:**
We thank the reviewer for this helpful comment. We reviewed the Results section to ensure that quotations were placed under appropriate themes and introduced with clearer interpretive framing. Where participants spoke generally about community norms rather than personal experiences, we adjusted the surrounding text to avoid overstating the quotation as a direct lived experience.

**Location of revision:**
Results section, particularly subsections “Individual-level barriers,” “Limited knowledge and awareness,” “Limited healthcare-seeking behavior,” “Stigmatization,” and “Health system-level barriers.”

**Reviewer comment 13:**
*Consider reducing repetition in Results, as some themes overlap.*

**Response:**
We thank the reviewer for this suggestion. We agree that some passages in the Results were repetitive, particularly where explanatory text repeated the content of participant quotations or where tracked revisions had left overlapping old and new wording. We conducted a light editorial revision to reduce repetition while preserving the participant-centred nature of the Results.

**Location of revision:**
Results section, especially subsections “Limited healthcare-seeking behavior,” “Limited leprosy expertise among healthcare workers,” and “Awareness campaigns.”

**Reviewer comment 14:**
*Ensure consistent terminology, e.g., “delay,” “barrier,” “pathway.”*

**Response:**
We thank the reviewer for this helpful recommendation. We have clarified the terminology used throughout the manuscript. We now define diagnostic delay as the period from first recalled symptom to diagnosis, diagnostic pathway as the sequence of symptom recognition, care-seeking, consultations, referrals, and eventual diagnosis, and barriers as factors that prolonged this pathway. We also reviewed the manuscript to improve consistency in the use of these terms.

**Location of revision:**
Methodology section, subsection “Data Collection and Analysis,” and terminology harmonized throughout the Results, Discussion, and Recommendations sections.
