# Supplementary material for: Delayed diagnosis as a barrier to Zero Leprosy: Lived experiences from Pakistan
Source: PLOS Glob Public Health. 2026 Aug 27;6(8):e0007184. doi: 10.1371/journal.pgph.0007184 (PMC13521354; doi:10.1371/journal.pgph.0007184)
Supplement: Peer review history

# Peer review: Decision Letter 0

**DOI:** 10.1371/journal.pgph.0007184.r001

**Authors:** Kanokwan Suwannarong

22 May 2026

PGPH-D-26-00426

Delayed diagnosis as a barrier to Zero Leprosy: lived experiences from Pakistan

PLOS Global Public Health

Dear Dr. Fastenau,

Thank you for submitting your manuscript to PLOS Global Public Health. After careful consideration, we feel that it has merit but does not fully meet PLOS Global Public Health’s publication criteria as it currently stands. Therefore, we invite you to submit a revised version of the manuscript that addresses the points raised during the review process.

Kindly note that all reviewers considered this manuscript to be well-positioned for publication in PLOS Global Public Health. However, several revisions are required, primarily related to:

- Enhancing the analytical depth
- Strengthening the theoretical engagement
- Clarifying the linkage between the findings and recommendations

Please submit your revised manuscript by Jul 21 2026 11:59PM. If you will need more time than this to complete your revisions, please reply to this message or contact the journal office at globalpubhealth@plos.org. When you’re ready to submit your revision, log on to https://www.editorialmanager.com/pgph/ and select the 'Submissions Needing Revision' folder to locate your manuscript file.

Please include the following items when submitting your revised manuscript:

- A letter that responds to each point raised by the editor and reviewer(s). You should upload this letter as a separate file labeled 'Response to Reviewers'.
- A marked-up copy of your manuscript that highlights changes made to the original version. You should upload this as a separate file labeled 'Revised Manuscript with Track Changes'.
- An unmarked version of your revised paper without tracked changes. You should upload this as a separate file labeled 'Manuscript'.

Guidelines for resubmitting your figure files are available below the reviewer comments at the end of this letter.

As the corresponding author, your ORCID iD is verified in the submission system and will appear in the published article. PLOS supports the use of ORCID, and we encourage all coauthors to register for an ORCID iD and use it as well. Please encourage your coauthors to verify their ORCID iD within the submission system before final acceptance, as unverified ORCID iDs will not appear in the published article. *Only*  the individual author can complete the verification step; PLOS staff *cannot*  verify ORCID iDs on behalf of authors.

We look forward to receiving your revised manuscript.

Kind regards,

Kanokwan Suwannarong, Ph.D.

Academic Editor

PLOS Global Public Health

Journal Requirements:

1. We note that the ethics approval documents should not be published with the paper and are for internal information only. Please change the file type to 'Other' to ensure that they are not published.

2. We have noticed that you have uploaded Supporting Information files, but you have not included a list of legends. Please add a full list of legends for your Supporting Information files before or after the references list.

3. Some material included in your submission may be copyrighted. According to PLOS’s copyright policy, authors who use figures or other material (e.g., graphics, clipart, maps) from another author or copyright holder must demonstrate or obtain permission to publish this material under the Creative Commons Attribution 4.0 International (CC BY 4.0) License used by PLOS journals. Please closely review the details of PLOS’s copyright requirements here: PLOS Licenses and Copyright. If you need to request permissions from a copyright holder, you may use PLOS's Copyright Content Permission form.

Please respond directly to this email or email the journal office and provide any known details concerning your material's license terms and permissions required for reuse, even if you have not yet obtained copyright permissions or are unsure of your material's copyright compatibility.

Potential Copyright Issues:

"Information Sheet.pdf" contains branding/a logo. We are not permitted to publish this under our CC-BY 4.0 license, even with permission. We ask that you please remove or replace it.

4. If the reviewer comments include a recommendation to cite specific previously published works, please review and evaluate these publications to determine whether they are relevant and should be cited. There is no requirement to cite these works unless the editor has indicated otherwise.

[Note: HTML markup is below. Please do not edit.]

Reviewers' comments:

Reviewer's Responses to Questions

**Comments to the Author**

1. Does this manuscript meet PLOS Global Public Health’s publication criteria?

Reviewer #1: Yes

Reviewer #2: Yes

Reviewer #3: Yes

Reviewer #4: Yes

**********

2. Has the statistical analysis been performed appropriately and rigorously?

Reviewer #1: Yes

Reviewer #2: N/A

Reviewer #3: Yes

Reviewer #4: Yes

**********

3. Have the authors made all data underlying the findings in their manuscript fully available (please refer to the Data Availability Statement at the start of the manuscript PDF file)?

The PLOS Data policy

Reviewer #1: No

Reviewer #2: Yes

Reviewer #3: Yes

Reviewer #4: Yes

**********

4. Is the manuscript presented in an intelligible fashion and written in standard English?

Reviewer #1: Yes

Reviewer #2: Yes

Reviewer #3: Yes

Reviewer #4: Yes

**********

Reviewer #1: This manuscript presents a valuable qualitative exploration of the barriers to timely leprosy diagnosis in Pakistan, a "low-endemic" setting that continues to face high rates of Grade 2 Disability (G2D) at the time of diagnosis. By focusing on the lived experiences of those already suffering from visible deformities, the authors provide critical insights into why "Zero Leprosy" remains elusive despite the availability of free treatment. The study is well-structured, ethically sound, and directly addresses the human and systemic failures that sustain hidden transmission.

1. Strengths

Human-Centric Perspective: Unlike many quantitative studies that focus solely on "delay intervals," this research prioritizes the narratives of affected individuals. This approach highlights the "human cost" of late detection and reveals gaps that surveillance data alone cannot capture.

The analysis successfully categorizes barriers into individual, community, and health system levels. This holistic view is essential for programmatic planning, as it shows that clinical expertise alone is insufficient if social stigma or financial "opportunity costs" prevent patients from reaching the clinic.

A significant strength is the inclusion of a dedicated section for participant-suggested strategies. The recommendation to utilize treated patients as "agents of change" and peer educators is a culturally grounded and empowering solution for reducing stigma and encouraging early care-seeking.

The use of thematic content analysis with ATLAS.ti 24 and the explicit mention of reaching data saturation at 19 interviews lend credibility to the qualitative findings.

The authors followed the Declaration of Helsinki and obtained approval from the Marie Adelaide Leprosy Centre (MALC) Ethical Review Committee. The use of thumbprints for participants with disabilities or limited literacy ensures inclusive ethical practice.

2. Areas for Improvement

Methodology & Data Handling

The authors note that non-simultaneous translation was used, meaning statements were "summarized rather than literally reproduced". In qualitative research, the exact phrasing often carries significant weight. While the authors took steps to minimize errors, this remains a limitation that could have impacted the "verbatim" nature of the transcriptions mentioned later. Recommendation: Discuss more explicitly how summarized translation might have affected the "richness" of the data and whether any back-translation or member-checking was used to ensure accuracy.

The sample consists of 14 males and only 5 females. While the manuscript discusses gender-specific barriers (e.g., the need for male chaperones and lack of female healthcare workers), the small number of female participants may limit the depth of these findings. Recommendation: Acknowledge this skew more clearly as a limitation and suggest that future research specifically target female populations to fully understand the intersection of gender and disability in this context.

Some participants reported delays of up to 30 years. While the authors mention recall bias, they should further discuss how the health system context in Pakistan 30 years ago differs from today. This would help clarify if the reported barriers are historical or persistent in the current landscape.

Data Availability Statement

The current statement says data is not available due to "sensitive information". While participant privacy is paramount in leprosy research, PLOS policies usually require the sharing of anonymized/de-identified data. Recommendation: Provide a more detailed justification for why de-identified transcripts cannot be made available in a repository, or clarify if a "minimal data set" can be generated.

Discussion & Contextualization

The manuscript frequently references "Zero Leprosy". The Discussion could be strengthened by more explicitly linking the findings to the three pillars of the Global Leprosy Strategy: zero transmission, zero disability, and zero discrimination. For example, highlight how "label-based stigma" specifically impedes the "zero discrimination" goal.

The paper notes that leprosy services are largely NGO-driven in Pakistan. The authors should expand on the policy implications of this. If the goal is "Zero Leprosy" by 2030, how can the government transition from a "passive" observer to a leader in integrating leprosy into the national primary healthcare system?

3. Technical Corrections

The manuscript mentions Pakistan is classified as "low endemic". It would be helpful to briefly provide the WHO threshold for this classification in the Introduction to orient readers unfamiliar with leprosy epidemiology.

While generally well-written, ensure that quotes rewritten to the "first person" (as mentioned in the Methods) do not inadvertently change the participant's original meaning or emphasis.

The authors correctly identify the absence of children in the study population as a limitation. Given that child leprosy cases are a primary indicator of recent transmission, emphasize this more strongly in the "Recommendations" for future research.

Reviewer #2: The manuscript makes a relevant and timely contribution to the field of global public health by exploring in depth the delay in leprosy diagnosis in Pakistan from the perspective of people diagnosed with grade 2 disability. The study is well aligned with the scope of PLOS Global Public Health and the principles of the Zero Leprosy agenda, highlighting the clinical, programmatic, social, and ethical implications of late diagnosis in low-endemicity contexts.

From a methodological point of view, the qualitative design is appropriate for the proposed objectives and was conducted rigorously. The eligibility criteria, the recruitment process, data collection through in-depth interviews, and thematic analysis are clearly described. The use of dedicated software for qualitative analysis, the combination of deductive and inductive coding, and the explicit statement of the saturation point lend transparency and credibility to the analytical process. The limitations of the study—including possible recall bias and challenges related to translation—are recognized and discussed adequately, which reinforces the ethical and reflective stance of the authors.

The conclusions presented are coherent and supported by the data, respecting the interpretative limits of a qualitative study. The findings are well anchored in the existing literature and broaden the understanding of how individual, community, and structural factors interact to prolong the time to diagnosis, resulting in preventable disability and perpetuation of transmission. The emphasis on lived experiences adds substantial value to the debate, complementing quantitative studies and analyses focused exclusively on the health system.

Regarding data availability, although the primary data are not accessible in a public repository, the justification presented—involving the sensitivity of the information and the protection of a vulnerable group—is adequate and consistent with the PLOS data policy for qualitative research. The clear indication of an institutional contact point for data requests, coupled with the presentation of representative excerpts from the interviews, allows for the assessment of the robustness of the analyses and the coherence between data and conclusions.

From an ethical standpoint, the study demonstrates full compliance with international standards, with approval by an ethics committee, obtaining informed consent, and appropriate confidentiality measures. There are no indications of concerns related to research ethics, publication ethics, or duplicate publication.

Regarding the manuscript's presentation, the text is clear, well-structured, and written in appropriate standard English. However, a light editorial review is recommended to improve conciseness and fluency in some sections, especially in the Results, which are at times lengthy and could be summarized without analytical loss. Minor language adjustments and reduction of repetitions can further increase the text's accessibility to non-specialist readers.

As a constructive suggestion, the authors could consider:

• reinforcing, in a more concise way in the Discussion, the link between diagnostic delay and concrete programmatic implications for health systems in low-endemicity countries;

• indicating more explicitly how the qualitative findings can inform future quantitative research or mixed studies, especially regarding the interruption of transmission.

In summary, this is a robust, well-founded manuscript of high scientific and programmatic value, which makes a significant contribution to the literature on leprosy, timely diagnosis, and global public health. I recommend its publication, subject only to any minor editorial adjustments.

Reviewer #3: Journal: PLOS Global Public Health

Manuscript ID: PGPH-D-26-00426

Title (inferred): Diagnostic Delay in Leprosy in Pakistan: Lived Experiences of Persons with Grade 2 Disability

Article Type: Qualitative Research

1. Overall Assessment

This manuscript presents a qualitative investigation of diagnostic delay in leprosy in Pakistan, drawing on lived experiences of individuals with grade 2 disability (G2D). The study provides rich insights into multi-level barriers (individual, community, and health system) contributing to delayed diagnosis and ongoing transmission.

The manuscript is highly relevant to global public health, particularly within the domains of:

• Neglected tropical diseases (NTDs)

• Health systems strengthening

• Stigma and health-seeking behavior

• Disease elimination strategies

The study is well-structured, analytically coherent, and policy-relevant, and makes a meaningful contribution to the “Zero Leprosy” discourse.

2. Significance and Contribution

The manuscript makes several important contributions:

• Positions diagnostic delay as a central driver of transmission, not just disability

• Integrates lived-experience perspectives, often underrepresented in programmatic research

• Highlights the paradox of low-endemic settings with high disability burden

• Provides actionable recommendations aligned with elimination strategies

The framing of diagnostic delay as a “systemic and cumulative failure” rather than a single-point issue is particularly compelling.

3. Major Comments

3.1 Strength of Conceptual Framing

The manuscript successfully links diagnostic delays to:

• Transmission dynamics

• Missed prevention opportunities

• Health system gaps

• Stigma and concealment

For example, the discussion clearly articulates that:

prolonged undiagnosed periods create repeated opportunities for transmission

This is a strong and policy-relevant argument.

Strength noted: The integration of Zero Leprosy framework is appropriate and well-developed.

3.2 Depth of Qualitative Analysis

The thematic analysis is generally robust, supported by participant quotations. However:

• At times, the manuscript leans toward descriptive reporting rather than deeper analytical interpretation.

• Some themes (e.g., stigma, gender barriers) could be more explicitly linked to existing theoretical frameworks (e.g., stigma theory, structural violence).

Recommendation:

Strengthen interpretive depth by:

• Engaging with theory

• Moving beyond “what participants said” to “what this means structurally”

3.3 Representation and Sampling

The study focuses exclusively on individuals with grade 2 disability, which is both a strength and a limitation.

• Strength: captures severe consequences of delay

• Limitation: excludes early-stage patients and caregivers

The manuscript acknowledges this limitation, but:

• It should more clearly discuss how this focus may bias findings toward extreme delay narratives.

3.4 Recall Bias and Time Estimation

Participants reported delays ranging from months to decades.

Given the retrospective nature:

• Recall bias is acknowledged but could be further emphasized.

• Consider discussing how memory reconstruction may influence delay estimates.

3.5 Health System Critique

The manuscript effectively identifies:

• Misdiagnosis

• Lack of clinical awareness

• Weak referral systems

However:

• The critique could be sharpened by distinguishing between:

o Primary care failures

o Specialist-level gaps

o Structural vs operational issues

3.6 Recommendations Section

The recommendations are comprehensive and well-aligned with findings, including:

• Awareness campaigns

• Patient involvement

• Training healthcare workers

• Strengthening referral pathways

• Gender-sensitive interventions

However:

• Some recommendations appear broad and programmatic rather than directly derived from qualitative insights.

Recommendation:

Explicitly link each recommendation to specific findings/themes.

4. Minor Comments

4.1 Terminology

• Replace “patients” with:

“persons affected by leprosy” (person-centered language)

4.2 Use of Quotations

• Quotations are appropriate and illustrative.

• Consider slightly reducing repetition and prioritizing the most analytically rich excerpts.

4.3 Gender Analysis

The manuscript highlights gender barriers (e.g., limited access to female healthcare workers) .

• This is important but could be expanded:

o Are delays systematically longer for women?

o How do gender norms shape concealment or care-seeking?

4.4 Ethical and Data Transparency

• Ethical approval and consent are clearly stated.

• Data availability is restricted due to sensitivity, which is appropriate.

4.5 Language and Style

• Overall well-written.

• Minor grammatical edits may improve flow in some sections.

Reviewer #4: Manuscript Title:

Delayed diagnosis as a barrier to Zero Leprosy: lived experiences from Pakistan

General Comments

This manuscript addresses a highly relevant public health issue, diagnostic delay in leprosy, and its implications for disability and ongoing transmission in Pakistan. The focus on individuals with grade 2 disability (G2D) provides important insight into late-stage diagnosis and contributes valuable patient-centered perspectives to the literature.

The qualitative approach is appropriate for exploring lived experiences, and the manuscript is generally well-written and conceptually grounded within the “Zero Leprosy” framework.

However, several methodological, analytical, and reporting issues require clarification and strengthening to enhance the scientific rigor, internal coherence, and interpretability of the findings.

Major Comments

1. Misalignment between objective, methods, and findings

The stated objective focuses on exploring reasons for delayed diagnosis. However, the methods and results indicate that the study also captured participants’ suggestions for improving services. This broader scope is not reflected in the objective.

The authors should revise the objective to clearly reflect all components of the study, including both:

• reasons for diagnostic delay, and

• proposed solutions or recommendations from participants

Ensuring alignment across the objective, methods, results, and discussion will improve the overall coherence of the manuscript.

2. Participant selection and recruitment

The process for identifying and recruiting study participants is insufficiently described.

• The manuscript notes that Pakistan reports approximately 250 new leprosy cases annually, with a significant proportion presenting with Grade 2 disability. However, it is unclear:

o how participants were sampled from this population

o What inclusion criteria were applied beyond disability grade

o how participants were approached and invited

• Additionally, the manuscript states that the year of diagnosis was not a restriction, implying inclusion of individuals diagnosed many years prior. It is unclear:

o how such participants were identified

o whether registries or facility records were used

Recommendation:

The authors should:

• Clearly describe sampling strategy and recruitment process

• Explain how older cases were traced

• Justify inclusion of long-term retrospective participants

• Clarify whether sampling aimed at saturation across key characteristics

3. Recall bias and temporal heterogeneity

Participants reported delays up to 30 years and diagnoses spanning decades.

This introduces:

• recall bias

• contextual inconsistency (the health system in 1989 vs 2024 is not comparable)

Currently, this is acknowledged but not adequately addressed analytically.

Recommendation:

• Stratify or reflect on differences between recent vs older diagnoses

• Discuss how changing health systems may influence findings

• Consider limiting interpretation or clearly framing findings as cross-generational narratives

5. Lack of reflexivity

There is no discussion of the researchers' positionality, which is expected in qualitative studies.

Missing elements:

• Who conducted interviews (insider vs outsider)?

• Relationship to MALC (potential influence on responses)?

• Power dynamics between the interviewer and participants

Recommendation:

Include a reflexivity statement, addressing:

• researcher background

• potential influence on data collection and interpretation

6. Conceptual inconsistency in the thematic framework

There appears to be an inconsistency in how themes are presented: The methods mention five broad themes, including “characteristics of the disease.” In the results, this appears embedded under “individual barriers.”

This weakens analytical clarity.

Recommendation:

• Present a clear and consistent thematic framework

• Consider adding a figure/table showing themes and subthemes

7. Definition and measurement of “diagnostic delay.”

The manuscript states: “All participants experienced diagnostic delay.” However, there is no provision of an operational definition of diagnostic delays. This weakens conceptual clarity; the author could define diagnostic delay explicitly (e.g., symptom onset → diagnosis) and explain how it was estimated or avoid presenting delay values if not systematically measured

8. Nature of participant narratives (experience vs perception)

Some quotes under the individual thematic area use “they” instead of “I”. This creates ambiguity: Were the participants sharing lived experiences or general community perceptions?

The author could clarify the distinction between personal experience vs perceived norms and ensure consistency in interpretation

9. Gender analysis is underdeveloped

The manuscript highlights gender barriers but relies heavily on female voices and does not mention how this compares with male participants.

10. Over-interpretation in Discussion

The Discussion is strong but somewhat overextended. It introduces transmission dynamics, policy implications, and Zero Leprosy framing, yet some of these are not directly grounded in the presented data

Recommendation: The author should strengthen the linkage between findings and interpretation. In addition, he should avoid introducing concepts not clearly supported by results

Minor Comments

1. Provide a clearer description of MALC’s role in the health system - The manuscript does not provide sufficient information about the MALC (presumably a leprosy center). The authors should clarify: the role of MALC within the health system, whether it is a referral center or primary diagnostic facility, and whether all leprosy patients in the region are managed there. This context is essential for understanding the representativeness of the study population.

2. Ensure all quotes align clearly with the themes they illustrate

3. Consider reducing repetition in Results (some themes overlap)

4. Ensure consistent terminology (e.g., “delay,” “barrier,” “pathway”)

Strengths of the Manuscript

Addresses an important neglected tropical disease with a strong focus on lived experiences, policy-relevant insights, rich qualitative data, and clear public health relevance

Overall Recommendation

The manuscript addresses a critical and underexplored aspect of leprosy control and provides valuable patient-centered insights. However, substantial revisions are required, particularly in:

• methodological transparency

• conceptual clarity

• alignment across sections

Addressing these issues will significantly strengthen the manuscript’s contribution to the field.

**********

what does this mean?). If published, this will include your full peer review and any attached files.

**Do you want your identity to be public for this peer review?**  If you choose “no”, your identity will remain anonymous but your review may still be made public.

For information about this choice, including consent withdrawal, please see our Privacy Policy

Reviewer #1: **Yes:**  Clara Abou Samra

Reviewer #2: No

Reviewer #3: **Yes:**  Abdulganiyyu A Ibrahim

Reviewer #4: No

**********

[NOTE: If reviewer comments were submitted as an attachment file, they will be attached to this email and accessible via the submission site. Please log into your account, locate the manuscript record, and check for the action link "View Attachments". If this link does not appear, there are no attachment files.]

# Author comment: Author response to Decision Letter 1

**DOI:** 10.1371/journal.pgph.0007184.r002

26 May 2026

Attachment Submitted filename: Point by Point Responses_PLOS.docx

# Peer review: Decision Letter 1

**DOI:** 10.1371/journal.pgph.0007184.r003

**Authors:** Kanokwan Suwannarong

31 Jul 2026

PGPH-D-26-00426R1

Delayed diagnosis as a barrier to Zero Leprosy: lived experiences from Pakistan

PLOS Global Public Health

Dear Dr. Fastenau,

Thank you for submitting your manuscript to PLOS Global Public Health. After careful consideration, we feel that it has merit but does not fully meet PLOS Global Public Health’s publication criteria as it currently stands. Therefore, we invite you to submit a revised version of the manuscript that addresses the points raised during the review process.

**Please revise Table 1 as follows:**

**Heading of Table 1: Should start with capital letters; for example: Participant, Age, Place of residence, Gender, Employment, Educational. For the education should be revised to “Educational Attainment Level”**

Please submit your revised manuscript by Aug 30 2026 11:59PM. If you will need more time than this to complete your revisions, please reply to this message or contact the journal office at globalpubhealth@plos.org. When you're ready to submit your revision, log on to https://www.editorialmanager.com/pgph/ and select the 'Submissions Needing Revision' folder to locate your manuscript file.

Please include the following items when submitting your revised manuscript:

- A letter that responds to each point raised by the editor and reviewer(s). You should upload this letter as a separate file labeled 'Response to Reviewers'.
- A marked-up copy of your manuscript that highlights changes made to the original version. You should upload this as a separate file labeled 'Revised Manuscript with Track Changes'.
- An unmarked version of your revised paper without tracked changes. You should upload this as a separate file labeled 'Manuscript'.

Guidelines for resubmitting your figure files are available below the reviewer comments at the end of this letter.

As the corresponding author, your ORCID iD is verified in the submission system and will appear in the published article. PLOS supports the use of ORCID, and we encourage all coauthors to register for an ORCID iD and use it as well. Please encourage your coauthors to verify their ORCID iD within the submission system before final acceptance, as unverified ORCID iDs will not appear in the published article. *Only*  the individual author can complete the verification step; PLOS staff *cannot*  verify ORCID iDs on behalf of authors.

We look forward to receiving your revised manuscript.

Kind regards,

Kanokwan Suwannarong, Ph.D.

Academic Editor

PLOS Global Public Health

**Journal Requirements:**

If the reviewer comments include a recommendation to cite specific previously published works, please review and evaluate these publications to determine whether they are relevant and should be cited. There is no requirement to cite these works unless the editor has indicated otherwise.

Please review your reference list to ensure that it is complete and correct. If you have cited papers that have been retracted, please include the rationale for doing so in the manuscript text, or remove these references and replace them with relevant current references. Any changes to the reference list should be mentioned in the rebuttal letter that accompanies your revised manuscript. If you need to cite a retracted article, indicate the article’s retracted status in the References list and also include a citation and full reference for the retraction notice.

**Reviewers' comments:**

Reviewer's Responses to Questions

**Comments to the Author**

Reviewer #1: All comments have been addressed

Reviewer #2: All comments have been addressed

Reviewer #3: All comments have been addressed

Reviewer #4: All comments have been addressed

**********

publication criteria?

Reviewer #1: Yes

Reviewer #2: Yes

Reviewer #3: Yes

Reviewer #4: Yes

**********

3. Has the statistical analysis been performed appropriately and rigorously?

Reviewer #1: Yes

Reviewer #2: N/A

Reviewer #3: Yes

Reviewer #4: Yes

**********

4. Have the authors made all data underlying the findings in their manuscript fully available (please refer to the Data Availability Statement at the start of the manuscript PDF file)?

The PLOS Data policy

Reviewer #1: Yes

Reviewer #2: Yes

Reviewer #3: Yes

Reviewer #4: Yes

**********

5. Is the manuscript presented in an intelligible fashion and written in standard English?

Reviewer #1: Yes

Reviewer #2: Yes

Reviewer #3: Yes

Reviewer #4: Yes

**********

**Reviewer #1:**  No additional comments

**Reviewer #2:**  This manuscript constitutes a valuable contribution to the literature on leprosy, delayed diagnosis, and strategies aimed at achieving the Zero Leprosy goal in low-endemicity settings. The study demonstrates adequate methodological rigor, high programmatic relevance, and considerable potential to inform public policies and strengthen leprosy surveillance, care, and control efforts.

The limitations inherent to the study are appropriately acknowledged and transparently discussed by the authors, and they do not undermine the robustness of the findings or the validity of the conclusions.

One of the manuscript’s major strengths lies in giving voice to people affected by leprosy and reconstructing their diagnostic trajectories from the perspective of lived experience. Although previous studies have explored factors associated with delayed diagnosis from the viewpoint of specialists and healthcare professionals, this work provides important additional evidence by focusing specifically on individuals who developed visible disabilities as a consequence of delayed diagnosis.

The study is particularly relevant because it demonstrates that diagnostic delay should not be viewed solely as a clinical problem, but rather as the result of a complex interplay of individual, social, and structural factors, including low risk perception, limited awareness of the signs and symptoms of the disease, social stigma, gender inequalities, structural weaknesses within the healthcare system, insufficient professional training, and shortcomings in referral and counter-referral systems. This perspective broadens the understanding of the phenomenon, highlights its multifactorial nature, and enhances the practical value of the findings for the design, implementation, and improvement of public policies aimed at leprosy control and elimination.

**Reviewer #3:**  Resubmission – Round 2

Manuscript ID: PGPH-D-26-00426

Title: Diagnostic Delay in Leprosy in Pakistan: Lived Experiences of Persons with Grade 2 Disability

Article Type: Qualitative Research

The authors have responded thoroughly to the major concerns raised during the previous review round. The revised manuscript is substantially improved, particularly in the areas of theoretical framing, interpretation of findings, limitations, and policy relevance. The manuscript now presents a coherent and compelling account of how diagnostic delay contributes not only to disability but also to ongoing transmission, thereby impeding progress toward Zero Leprosy goals. The study offers valuable insights from persons affected by leprosy and makes a meaningful contribution to global public health and NTD literature.

Most remaining issues are editorial and relate to strengthening methodological transparency and ensuring consistency in interpretation.

Major Strengths of the Revision

1. Improved Theoretical and Analytical Depth

The revised discussion now goes beyond descriptive reporting and incorporates concepts such as:

• Stigma as a multi-level phenomenon

• Structural violence

• Health system failures at multiple levels

• Transmission dynamics in low-endemic settings

These additions substantially strengthen the manuscript's contribution to global health scholarship. The discussion of stigma operating at interpersonal, anticipatory, and internalized levels is particularly effective.

2. Stronger Linkage to Zero Leprosy Framework

The manuscript now clearly positions diagnostic delay as a barrier to:

• Zero transmission

• Zero disability

• Zero discrimination

This framing enhances the policy relevance of the findings and aligns well with current global leprosy strategies.

3. Improved Limitations Section

The revised limitations section appropriately acknowledges:

• Recall bias

• Memory reconstruction

• Translation challenges

• Gender imbalance

• Focus on G2D participants only

This significantly improves the manuscript's credibility and transparency.

4. Better Alignment Between Findings and Recommendations

The recommendations are now explicitly linked to themes emerging from participant narratives, which addresses a key concern from the previous review.

Remaining Issues

1. Reflexivity and Researcher Positionality

Although methodological reporting has improved, the manuscript still lacks sufficient discussion of researcher reflexivity.

The authors should briefly address:

• Interviewer backgrounds

• Relationship between researchers and participants

• Potential influence of professional affiliations with leprosy services

• Steps taken to minimize interpretive bias

This is particularly important in qualitative research involving stigmatized conditions.

Suggested action: Add a short paragraph within the Methods section addressing reflexivity.

2. Trustworthiness of the Analysis

The manuscript would benefit from more explicit reporting of qualitative rigor procedures.

Please clarify:

• Whether thematic saturation was assessed and how it was determined.

• Whether coding was performed independently by more than one researcher.

• How coding disagreements were resolved.

• Whether any form of peer debriefing or audit trail was maintained.

These additions would strengthen methodological transparency.

3. Sampling and Transferability

The manuscript acknowledges that all participants had grade 2 disability, which is appropriate. However, a brief statement should be added to emphasize that the findings may not fully reflect the experiences of:

• Individuals diagnosed earlier

• Persons without disability

• Individuals who have never reached specialized services

Although partly discussed in limitations, a clearer statement regarding transferability would be beneficial.

4. Language and Terminology

A few instances still use provider-centered terminology, such as:

"patients"

Consider consistently using:

"persons affected by leprosy"

throughout the manuscript to align with contemporary person-centered language in NTD research.

Minor Editorial Comments

Discussion

The discussion is now comprehensive; however, a small amount of condensation could improve readability. Some sections repeat similar arguments regarding transmission and delayed diagnosis.

Recommendations

The recommendations section is strong, but the proposed hotline for healthcare workers and information desk concepts could be briefly identified as participant-generated suggestions rather than evidence-based interventions.

Data Availability Statement

The revised data availability statement is thoughtful and appropriately balances transparency with confidentiality concerns. No further revisions required.

**Reviewer #4** : I have carefully reviewed the revised version and the authors' responses to the reviewers' comments. The authors have adequately addressed the concerns raised in the initial review and have made appropriate revisions throughout the manuscript. The objectives, methods, results, and discussion are now presented more clearly, and the manuscript has been strengthened considerably.

I have no further major comments and believe the manuscript is suitable for publication.

**********

what does this mean?). If published, this will include your full peer review and any attached files.

**Do you want your identity to be public for this peer review?**  If you choose “no”, your identity will remain anonymous but your review may still be made public.

For information about this choice, including consent withdrawal, please see our Privacy Policy

Reviewer #1: **Yes:**  Clara Abou Samra

Reviewer #2: **Yes:**  Alexandre Casimiro de Macedo

Reviewer #3: **Yes:**  Dr. Abdulganiyyu A. Ibrahim

Reviewer #4: **Yes:**  Rhoda Pola

**********

[NOTE: If reviewer comments were submitted as an attachment file, they will be attached to this email and accessible via the submission site. Please log into your account, locate the manuscript record, and check for the action link "View Attachments". If this link does not appear, there are no attachment files.]

**Figure Resubmissions:**

# Author comment: Author response to Decision Letter 2

**DOI:** 10.1371/journal.pgph.0007184.r004

31 Jul 2026

Attachment Submitted filename: Response to the Editor and Reviewers_PGPH_310726.pdf

# Editor report: Decision Letter 2

**DOI:** 10.1371/journal.pgph.0007184.r005

**Authors:** Kanokwan Suwannarong

5 Aug 2026

PGPH-D-26-00426R2

Delayed diagnosis as a barrier to Zero Leprosy: lived experiences from Pakistan

PLOS Global Public Health

Dear Dr. Fastenau,

Thank you for submitting your manuscript to PLOS Global Public Health. After careful consideration, we feel that it has merit but does not fully meet PLOS Global Public Health’s publication criteria as it currently stands. Therefore, we invite you to submit a revised version of the manuscript that addresses the points raised during the review process.

Please submit your revised manuscript by Sep 04 2026 11:59PM. If you will need more time than this to complete your revisions, please reply to this message or contact the journal office at globalpubhealth@plos.org. When you're ready to submit your revision, log on to https://www.editorialmanager.com/pgph/ and select the 'Submissions Needing Revision' folder to locate your manuscript file.

Please include the following items when submitting your revised manuscript:

- A letter that responds to each point raised by the editor and reviewer(s). You should upload this letter as a separate file labeled 'Response to Reviewers'.
- A marked-up copy of your manuscript that highlights changes made to the original version. You should upload this as a separate file labeled 'Revised Manuscript with Track Changes'.
- An unmarked version of your revised paper without tracked changes. You should upload this as a separate file labeled 'Manuscript'.

Guidelines for resubmitting your figure files are available below the reviewer comments at the end of this letter.

As the corresponding author, your ORCID iD is verified in the submission system and will appear in the published article. PLOS supports the use of ORCID, and we encourage all coauthors to register for an ORCID iD and use it as well. Please encourage your coauthors to verify their ORCID iD within the submission system before final acceptance, as unverified ORCID iDs will not appear in the published article. *Only*  the individual author can complete the verification step; PLOS staff *cannot*  verify ORCID iDs on behalf of authors.

We look forward to receiving your revised manuscript.

Kind regards,

Kanokwan Suwannarong, Ph.D.

Academic Editor

PLOS Global Public Health

Journal Requirements:

If the reviewer comments include a recommendation to cite specific previously published works, please review and evaluate these publications to determine whether they are relevant and should be cited. There is no requirement to cite these works unless the editor has indicated otherwise.

Please review your reference list to ensure that it is complete and correct. If you have cited papers that have been retracted, please include the rationale for doing so in the manuscript text, or remove these references and replace them with relevant current references. Any changes to the reference list should be mentioned in the rebuttal letter that accompanies your revised manuscript. If you need to cite a retracted article, indicate the article’s retracted status in the References list and also include a citation and full reference for the retraction notice.

Additional Editor Comments (if provided):

[Note: HTML markup is below. Please do not edit.]

Reviewers' comments:

[NOTE: If reviewer comments were submitted as an attachment file, they will be attached to this email and accessible via the submission site. Please log into your account, locate the manuscript record, and check for the action link "View Attachments". If this link does not appear, there are no attachment files.]

# Author comment: Author response to Decision Letter 3

**DOI:** 10.1371/journal.pgph.0007184.r006

5 Aug 2026

Attachment Submitted filename: Response_to_the_Editor_and_Reviewers_PGPH_310726_auresp_3.pdf

# Editor report: Decision Letter 3

**DOI:** 10.1371/journal.pgph.0007184.r007

**Authors:** Kanokwan Suwannarong

10 Aug 2026

Delayed diagnosis as a barrier to Zero Leprosy: lived experiences from Pakistan

PGPH-D-26-00426R3

Dear Dr. Fastenau,

We are pleased to inform you that your manuscript 'Delayed diagnosis as a barrier to Zero Leprosy: lived experiences from Pakistan' has been provisionally accepted for publication in PLOS Global Public Health.

Before your manuscript can be formally accepted you will need to complete some formatting changes, which you will receive in a follow up email. A member of our team will be in touch with a set of requests.

Please note that your manuscript will not be scheduled for publication until you have made the required changes, so a swift response is appreciated.

IMPORTANT: The editorial review process is now complete. PLOS will only permit corrections to spelling, formatting or significant scientific errors from this point onwards. Requests for major changes, or any which affect the scientific understanding of your work, will cause delays to the publication date of your manuscript.

If your institution or institutions have a press office, please notify them about your upcoming paper to help maximize its impact. If they'll be preparing press materials, please inform our press team as soon as possible -- no later than 48 hours after receiving the formal acceptance. Your manuscript will remain under strict press embargo until 2 pm Eastern Time on the date of publication. For more information, please contact globalpubhealth@plos.org.

Thank you again for supporting Open Access publishing; we are looking forward to publishing your work in PLOS Global Public Health.

Best regards,

Kanokwan Suwannarong, Ph.D.

Academic Editor

PLOS Global Public Health

***********************************************************

Reviewer Comments (if any, and for reference):
